# Synthesis, Biological Evaluation and Stability Studies of Some Novel Aza-Acridine Aminoderivatives

**DOI:** 10.3390/molecules25194584

**Published:** 2020-10-08

**Authors:** Maria Karelou, Vasileios Kourafalos, Athanasia P. Tragomalou, Panagiotis Marakos, Nicole Pouli, Ourania E. Tsitsilonis, Evangelos Gikas, Ioannis K. Kostakis

**Affiliations:** 1 Department of Pharmacy, Division of Pharmaceutical Chemistry, National and Kapodistrian University of Athens, Panepistimiopolis, Zografou, 15771 Athens, Greece; marykar@pharm.uoa.gr (M.K.); vkourafalos@gmail.com (V.K.); marakos@pharm.uoa.gr (P.M.); pouli@pharm.uoa.gr (N.P.); 2 Section of Animal & Human Physiology, Department of Biology, National & Kapodistrian University of Athens, Panepistimiopolis, Ilissia, 15771 Athens, Greece; athantra@med.uoa.gr (A.P.T.); rtsitsil@biol.uoa.gr (O.E.T.); 3 Laboratory of Analytical Chemistry, Department of Chemistry, National and Kapodistrian University of Athens, Panepistimiopolis, Zografou, 15771 Athens, Greece

**Keywords:** acridines, cancer, drug discovery, stability, computational chemistry, mass spectrometry

## Abstract

Several new amino-substituted aza-acridine derivatives bearing a basic side chain have been designed and synthesized. The antiproliferative activity of the target compounds has been evaluated against three cancer cell lines—namely HCT-116 (colorectal), the uterine sarcoma MES-SA, and its doxorubicin-resistant variant MES-SA/Dx5. A limited number of the new acridines showed marginal cytotoxicity against the tested cell lines; nevertheless, these analogues possessed a similar substitution pattern. The moderate biological activity of these derivatives was attributed to their instability in aqueous media, which has been studied by mass spectrometry and computational chemistry experiments at the density functional level of theory (DFT).

## 1. Introduction

Acridine derivatives have been extensively studied due to their wide range of biological activities and pharmaceutical properties. Regarding their anticancer activities, substantial efforts have been directed at designing and synthesizing novel compounds with improved pharmacological and toxicological profiles. Acridines are characterized by a unique, semi-planar, and hydrophobic scaffold that interacts with diverse biomolecular targets. These properties are mainly attributed to the effectiveness of the π conjugated structure to intercalate between double-stranded DNA, thereby inhibiting DNA replication in the rapidly growing cancer cells [1,2,3,4], However, intercalation alone cannot fully support the cytotoxicity shown by acridine derivatives; thus, the inhibition of various DNA-related enzymes such as topoisomerases [1,5], telomerases [6], and poly(ADP-ribose)polymerases (PARPs) [7], is implicated in their mode of action.

It has been reported that the effectiveness of acridin-9-amines is related to their ability to exist in two tautomeric forms; the heterocyclic nitrogen adopts an acceptor or donor conformation, which has a radical effect on the binding properties of the molecule [8,9]. On the other hand, structure–activity relationship (SAR) research indicates that the substitution with a 7-methoxy group, and/or an electron-deficient nitro group are crucial for their activity and selectivity. Furthermore, the incorporation of a flexible dialkylaminoalkylamino-substituted side chain results in a noticeable increase in potency. The distance between the two amino groups is essential and the optimal length is equal to two or three methylene units. Interestingly, if the ability of the amino group to participate in hydrogen bonding is hindered, the antitumor activity is significantly reduced or demolished [10,11,12].

**Figure 1:**
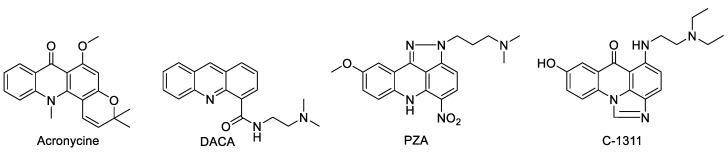
Structures of acronycine, DACA, PZA and C-1311.

In order to further improve the pharmacodynamic and pharmacokinetic properties of acridines and develop more efficient analogues, several structural modifications have been reported. Many of these derivatives exhibited noticeable cytotoxic activity and many have been proved to be clinically effective. The most characteristic examples include pyrazoloacridines [10,13,14,15], thiadiazinoacridines [12], triazoloacridones [16], and imidazoloacridones [17]; among them, the pyrazoloacridine PZA [13,14], an amino-substituted 5-nitropyrazolo [3,4,5-kl] acridine derivative, the imidazoloacridine C-1311 [11,18], the acridine carboxamide DACA [4], and the natural product acronycine [19,20] are of great interest (Figure 1). 

Although considerable progress has been made in this field, more work is needed to further improve the potency and selectivity of these compounds. In the course of our involvement in this area, we had previously studied several amino-substituted acridine derivatives with interesting anticancer properties [15,21,22,23]. These compounds share common structural features with the compounds described above, bearing a basic side chain and a nitro group, and exhibit substantial cytotoxic activities against a panel of cancer cell lines, presumably due to DNA binding and intercalation.

In an effort to explore the optimal structural requirements for acridines, in order to express biological activity and since there are only limited examples of acridines with an additional nitrogen participating in the ring junction, we designed a number of new amino-substituted aza-acridines and present herein the study of their synthesis, biological evaluation and stability. The rationale for this modification was prompted by the correlation of these compounds with potent acridines. In the new derivatives, the orientation of the amino chain substitution is strictly defined due to the strong intramolecular hydrogen bond between the acridone carbonyl and the adjacent amino group. On the other hand, due to the ring-junction nitrogen, the resulting scaffold is locked in one of the tautomeric forms, in which the acridine nitrogen adopts only the acceptor conformation. This alteration could induce a radical effect on the binding properties of the molecule.

## 2. Results and Discussion

### 2.1. Chemistry

**Scheme 1:**
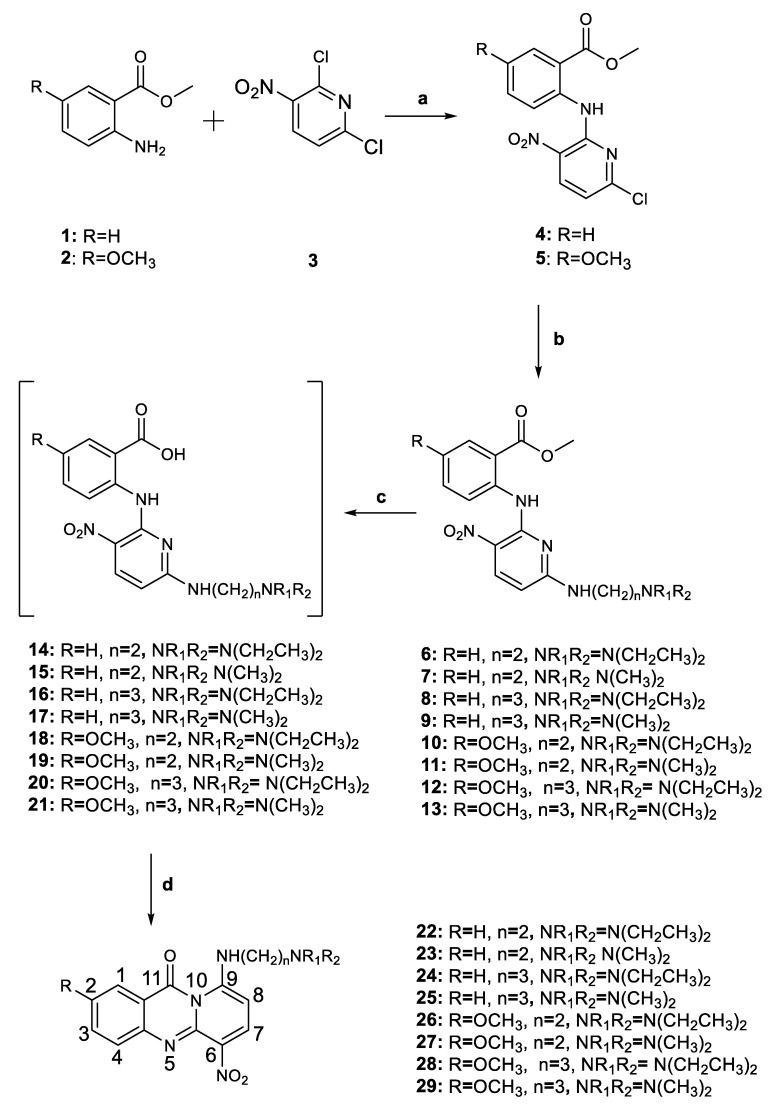
Reagents and conditions: a: NaH, THF dry, 60 °C, 2 h; b: suitable amine, ethanol abs., reflux, 12 h; c: NaOH 20%, MeOH, room temperature, 14 h; d: CF_3_COOH, (CF_3_CO)_2_O, 60 °C, 6 h.

Commercially available methyl anthranilate (**1**) or methyl 5-methoxyanthranilate (**2**) were used as starting materials (Scheme 1). Treatment of esters 1 and 2 with 2,6-dichloro-3-nitropyridine (**3**) afforded only the desired methyl esters **4** and **5**, respectively. The structure of these derivatives was elucidated using ^1^H and ^13^C NMR spectral data, using both direct and long-range homonuclear and heteronuclear correlation experiments (HMBC, HMQC, and NOESY sequences). Each one of the esters **4** and **5** was then reacted with the appropriately substituted *N*,*N*-dialkylethylenediamines or *N*,*N*-dialkylpropylenediamines providing the target amines **6**–**13**. Mild saponification of the esters **6**–**13** resulted in the carboxylic acids **14**–**21**, respectively, which, without further purification, were ring closed upon treatment with a 1/2 *v*/*v* mixture of trifluoroacetic anhydride and trifluoroacetic acid, providing the title compounds **22**–**29**.

Each compound’s structure was unambiguously established by ^1^H and ^13^C NMR spectroscopy, using both direct and long-range heteronuclear correlation experiments. Structural discrimination resulted from the observation that H-8 of the aza-acridines **22**–**29** exhibits ^4^*J* coupling with the carbonyl group, while in the case of esters **6**–**13** such a correlation is not possible. In general, the ^1^H NMR spectra of the esters 6–13 as well as the aza-acridines **22**–**29** showed slight differences, especially in the chemical shifts of the pyridine moiety. H-7 appears downfield as a doublet at approximately 8.30 ppm, while H-8 shifts upfield around 5.50 ppm.

### 2.2. Biological Evaluation

The in vitro antiproliferative activities of the new aza-acridine aminoderivatives were evaluated against three human tumor cell lines—namely, HCT-116 (colorectal), the uterine sarcoma MES-SA and its variant MES-SA/Dx5, reported to be 100 times more resistant to doxorubicin (Dx) [24]. The results of the MTT dye reduction assay, expressed as 50% inhibitory concentrations (IC_50_) in μM, are summarized in Table 1. For comparison, Dx and mitoxantrone (Mitox) were used as positive controls.

Most of the new compounds showed low cytotoxic activity and only three of them, namely **26**, **27** and **29**, were detected to possess a certain degree of cytotoxic activity which is enhanced against the two uterine sarcoma cell lines (MES-SA and MES-SA/Dx5). Even so, it is noticeable that all three compounds possess the methoxy substitution, providing evidence that the presence of the methoxy group is favorable for the cytotoxicity of this class of compounds. This finding is consistent with previous observations [2,11,12] concerning the improved cytotoxic activity of methoxy-substituted analogues.

From a direct comparison of the cytotoxic activity against the Dx-sensitive (MES-SA) and Dx-resistant (MES-SA/Dx5) cell lines, it is observed that derivatives **22**–**25** exhibit equal cytotoxicity against both cell lines, as indicated by the relevant resistant factor (RF) values (~1.0). On the contrary, the methoxy-substituted compounds **26**–**29**, proved to be more active against the Dx-resistant (MES-SA/Dx5) cell line and thus retain full antiproliferative activity against P-glycoprotein-overexpressing cells, with a relevant resistant factor of ~0.5. It must be pointed that even if the overall activity of the derivatives is moderate, the potency of compounds **26**–**29** is enhanced against the two uterine sarcoma cell lines (MES-SA and MES-SA/Dx5). This probably indicates that the methoxy-substituted derivatives could overcome Multi Drug Resistance (MDR).

**Scheme 2:**
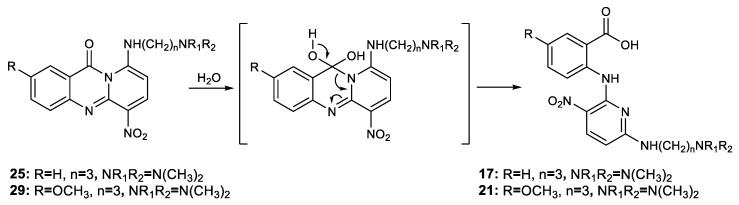
Hydrolysis of compounds **25** and **29**.

Overall, the activity of the new compounds was remarkably reduced when compared to the structurally related lead compounds (Figure 1). This is most likely due to the insertion of the additional nitrogen atom, which blocks the tautomerism of the acridine scaffold and could also interfere with the strength of the intramolecular hydrogen bond. On the other hand, it has been reported that aza-acridines are amenable to ring-opening [25] and we considered that the diminished activity of the new aza-acridines could potentially be due to hydrolytic instability. A hydrolysis mechanism should involve the nucleophilic attack of H_2_O to the carbonyl group, with concomitant ring-opening resulting in their precursor acids (**14**–**21**, Scheme 2). Therefore, the observed antiproliferative activity could be attributed to a mixture of aza-acridines and ring-opened compounds.

In order to verify our hypothesis, we performed a series of computational chemistry- and mass spectrometry-based hydrolysis experiments. Two compounds of the synthesized set of derivatives were selected as representative examples; i.e., **25** and its methoxy-substituted analogue **29**, aiming to evaluate the effect of the methoxy group on the stability and therefore on the activity.

### 2.3. Mass Spectrometry

**Figure 2:**
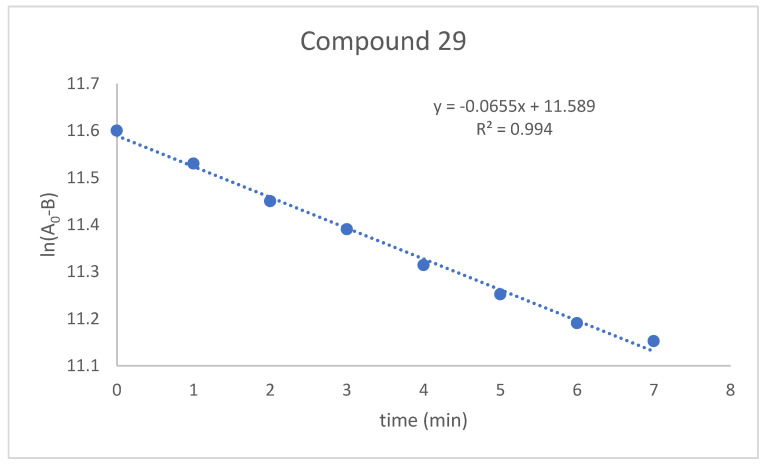
Pseudo first-order degradation of compound **29.**

In order to reveal the stability of compounds **25** and **29**, an initial estimation of the corresponding hydrolysis rates was performed. Both derivatives were found to be very unstable in an aqueous environment, albeit with different rates of hydrolysis, with the methoxy derivative **29** being far more stable. The results are presented in Figure 2.

In order to assimilate the conditions used for the biological testing of the substances, the substances were analyzed at the 20 μg/mL level (~60 μΜ). It should be pointed out that the use of mass spectrometry was chosen since the low concentration could make detection through NMR spectroscopy difficult. Furthermore, such a concentration is expected not to induce any matrix effect. Due to the fact that the hydrolysis products (i.e., **17** and **21**) afforded much cleaner spectra, the negative ion mode was employed. The kinetic equation used has been transformed to fit the data linearly. Thus, the equation used was

ln[A_0_ − B] = A_0_ − kt

where A_0_ is the initial concentration of each molecule, B the concentration of the respective product after hydrolysis and k is the hydrolysis constant. The two hydrolysis reactions were evaluated as linear models, based on the equation shown above. The statistical evaluation is presented in Table 2. The results are consistent with a first-order degradation reaction, as the fitting employing this equation order is linear. Rapid degradation was observed for the non-methoxy derivative under the conditions examined, whereas substantial degradation was observed for the methoxy analogue. Comparing the two slopes, which are the degradation coefficients, it was found that the degradation of **25** is **26** times faster than the corresponding hydrolysis of the methoxy derivative 29. Assuming that the reactions are pseudo first-order the corresponding t1/2 values are 10.6 min^−1^ for **25** and 277 min^−1^ for **29**.

### 2.4. Computational Chemistry

The two derivatives exhibit roughly the same geometry features. As the region of interest is located at the internal amide bond, the geometry features of the involved bonds are presented in Table 3. Both structures are planar, denoting a high aromaticity degree, whereas the bond lengths are essentially the same. Therefore, the difference in reactivity should be revealed by the electronic properties of the two molecules. Calculation of the electrostatic potential (ESP) (see Supplementary Materials, Figure S1) shows that the carbonyl C is depleted of electronic charge and therefore it is susceptible to an electrophile attack, which is consistent with the first step of hydrolysis (nucleophilic attack of water). Nevertheless, no appreciable difference has been observed between the two ESP surfaces, denoting that the differences calculated by the ESP methodology are too subtle to be detected.

**Figure 3:**
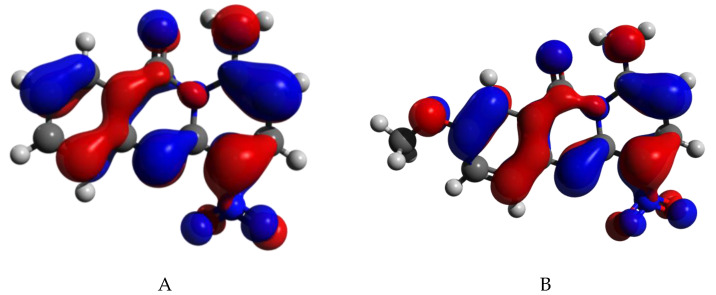
The HOMO orbitals of the non-methoxy-substituted analogue (**A**) and the methoxy-substituted analogue (**B**).

The electronic charge on carbonyl C as well as that of the connected N belonging to the internal amide bond also act as a measure of reactivity of the two derivatives. The Merz-Kollmann (MK) ESP-fitting atomic charges have been employed to estimate the electron partitioning scheme and showed that carbonyl C of the derivatives is the second most electron deficient carbon of the molecule with a charge of 0.761, slightly less than the pyridine carbon that is connected to the amine group, which bears a charge of 0.763. The attachment of the methoxy group renders carbonyl C the most electrophilic site of the molecule with a charge of 0.828 with the aforementioned pyridine carbon having an atomic charge of 0.797. Furthermore, the bond order of the C-N bond could also help explain the reactivity difference. The Mayer bond order analysis scheme that has been employed showed that the bond order increased by nearly 0.07 to the methoxy-bearing molecule, rendering the derivative more stable and thus resistant to hydrolysis. The delocalization of the HOMO orbital over the molecular skeleton provides visual assessment of the bonding across the atoms (Figure 3A,B). The spread of electronic density across the C-N bond of the internal amide in the case of the methoxy derivative indicates that the bond exhibits a higher overlap between the atomic orbitals (AOs) and therefore should be less prone to hydrolysis. The ELF function has been calculated for both molecules and the results can be visualized in Supplementary Figures S2A and S2B. In order to evaluate the concentration of electronic density the ELF value at the CP of the C-N bond has been assessed and was found to be 0.898 in both cases. Values larger than 0.7 indicate substantial π-character, but the ELF theory was not able to differentiate between the two molecules. Furthermore, the condensed Fukui function analysis was employed in order to pinpoint the electrophilic reactivity difference of the two carbonyl carbon atoms. The corresponding values were found to be 0.0207 and 0.0211 for the non-methoxy and methoxy derivative, respectively. The slight difference observed does not reflect their hydrolytic stability. Furthermore, as expected, their electrophilicity indices (e*eV) are also substantially similar (0.066 and 0.065, respectively). Interestingly, the condensed local softness (Hartree*e) for electrophilic attack is substantially different between the species. Thus, the corresponding values are 0.0874 for the non-methoxy derivative and 0.0933 for its methoxy counterpart. It is noteworthy that the computed local electrophilicity indexes (based on the Frozen Molecular Orbitals (FMOs) approximation) are also substantially different with values of 0.9608 and 0.7619 for the two molecules, but their order is opposite to the hydrolysis rate showing that this approach is not consistent with the experimental results in this case. Figure S3a,b shows the distribution of the Fukui function between the two molecules. Finally, Bader’s QTAIM (quantum theory of atoms-in-molecules) theory has been employed in order to study the electronic density partitioning between atoms. The AIM charges were calculated as well as the electron density Rho at the bond critical point connecting the carbonyl C to the pyridine N. The values obtained are for the carbon and nitrogen—+0.991 and −0.911, respectively—whereas for the BCP (bond critical point) a value of +0.253 was obtained for the non-methoxylated molecule. The corresponding values were +0.997, −0.911 and +0.255 for the methoxy derivative. The partitioning scheme does not indicate appreciable differences between the two molecular species. Finally, in order to estimate the energy difference between each molecule and its hydrolyzed counterpart, a full geometry optimization with the same level of theory has been performed. The corresponding values are −568.95 kcal/mol for the non-methoxylated derivative and -616.91 kcal/mol for its hydrolyzed derivative leading to an energy difference of 47.96 kcal/mol. The corresponding values for the methoxy molecule are −640.81 and -688.76 kcal/mol, leading to an energy difference of 47.95 kcal/mol. These results show that the thermodynamic result of the reaction is negligible and thus could not be the driving force of the more extensive degradation of the methoxy-bearing molecule. Overall it seems that the differentiation of the carbonyl carbon electronic charge as also depicted by the shape of the HOMO orbital could explain the difference of the hydrolytic stability, whereas the aromatic character as calculated by the topological analysis is not affected to a large extent.

## 3. Materials and Methods 

### 3.1. General Information

All commercially available chemicals and solvents were used as received without any further purification. Melting points were determined on a Büchi apparatus and are uncorrected. ^1^H NMR spectra and 2D spectra were recorded on a Bruker Avance DRX 400 instrument (Bruker BioSpin GmbH, Rheinstetten, Germany), whereas ^13^C NMR spectra were recorded on a Bruker AC 200 spectrometer (Bruker BioSpin GmbH, Rheinstetten, Germany) in deuterated solvents and were referenced to TMS (δ scale). The signals of ^1^H and ^13^C spectra were unambiguously assigned by using 2D NMR techniques: ^1^H-^1^H COSY, NOESY, HMQC, and HMBC. Mass spectra were recorded with a LTQ Orbitrap Discovery instrument (Thermo Scientific, Brehmen, Germany), possessing an Ionmax ionization source. Flash chromatography was performed on Merck silica gel 60 (0.040–0.063 mm) (Merck KGa, Darmstadt, Germany). Analytical thin layer chromatography (TLC) was carried out on precoated (0.25 mm) Merck silica gel F-254 plates. Stock solutions of compounds for biological experiments were prepared in DMSO (St. Louis, MO, USA) and stored at 20 °C. Working dilutions contained up to 0.1% *v*/*v* DMSO.

### 3.2. Synthesis

*Methyl 2-((6-chloro-3-nitropyridin-2-yl)amino)benzoate* (**4**): NaH (460 mg, 11.5 mmol, 60% in mineral oil) was added to a solution of methyl-2-aminobenzoate (1.63 g, 10.78 mmol, **1**) in dry THF (100 mL) at 0 °C under argon and the resulting suspension was stirred at room temperature for 30 min, followed by dropwise addition of a 2,6-dichloro-3-nitropyridine (2.26 g, 10.78 mmol, **3**) solution in dry THF (10 mL). The reaction mixture was then stirred at 60 °C for 2 h; an excess of NaH was quenched with methanol and the solvents were vacuum evaporated. The residue was dissolved in CH_2_Cl_2_, washed with a 10% Na_2_CO_3_ solution and water, dried over Na_2_SO_4_, and the solvent was evaporated to dryness. Flash chromatography on silica gel, using a mixture of cyclohexane–CH_2_Cl_2_ 3:1 as the eluent, afforded the title compound (2.22 g, 67 %) as an oil. ^1^H NMR (400 MHz, CDCl_3_) δ 12.46 (brs, 1H, D_2_O exch., NH), 8.54 (d, *J* = 8.6 Hz, 1H, H-4’), 8.50 (dd, *J* = 8.5, 1.1 Hz, 1H, H-3), 8.08 (d, *J* = 7.82 Hz, 1H, H-6), 7.59 (t, *J* = 7.82 Hz, 1H, H-4), 7.18 (t, *J* = 7.82 Hz, 1H, H-5), 6.87 (d, *J* = 8.61 Hz, 1H, H-5’), 3.98 (s, 3H, CH_3_). ^13^C NMR (50 MHz, CDCl_3_) δ 167.6 (C=O), 155.1 (C-2’), 148.5 (C-6’), 139.6 (C-2), 138.0 (C-4’), 133.5 (C-4), 131.2 (C-6), 128.8 (C-3’), 123.4 (C-3), 122.3 (C-5), 118.6 (C-1), 114.7 (C-5’), 52.5 (CH_3_). HR-MS (ESI) *m*/*z*: calcd. for C_13_H_11_ClN_3_O_4_^+^: [M1 + H]^+^ = 308.0433, found 308.0438.

*Methyl 2-((6-chloro-3-nitropyridin-2-yl)amino)-5-methoxybenzoate* (**5**): This compound was prepared by an analogous procedure as described for the preparation of **4** using compound **2**. Yield: 76 %. Oil. ^1^H NMR (400 MHz, CDCl_3_) δ 12.11 (brs, 1H, D_2_O exch., NH), 8.48 (d, *J* = 8.76 Hz, 1H, H-4’), 8.38 (d, *J* = 9.19 Hz, 1H, H-3), 7.55 (d, *J* = 3.01 Hz, 1H, H-6), 7.15 (dd, *J* = 9.19, 3.02 Hz, 1H, H-4), 6.81 (d, *J* = 8.76 Hz, 1H, H-5’), 3.98 (s, 3H, COOCH_3_), 3.86 (s, 3H, OCH_3_). ^13^C NMR (50 MHz, CDCl_3_) δ 167.1 (C=O), 155.4 (C-5), 155.3 (C-2’), 148.6 (C-6’), 138.0 (C-4’), 132.6 (C-2), 128.2 (C-3’), 124.4 (C-3), 120.1 (C-1), 119.8 (C-4), 114.9 (C-6), 114.1 (C-5’), 55.6 (OCH_3_), 52.5 (COOCH_3_). HR-MS (ESI) *m*/*z*: calcd. for C_14_H_13_ClN_3_O_5_^+^: [M1 + H]^+^ = 338.0538, found 338.0545.

*Methyl 2-((6-((2-(diethylamino)ethyl)amino)-3-nitropyridin-2-yl)amino)benzoate* (**6**): To a solution of chloride **4** (200 mg, 0.65 mmol) in absolute ethanol (20 mL) was added *N*,*N*-diethylethylenediamine (0.36 mL, 2.6 mmol) and the mixture was refluxed for 12 h. Upon cooling, the mixture was vacuum-evaporated, extracted with ethyl acetate-water, the organic layer was dried (Na_2_SO_4_) and evaporated to dryness. The residue was purified by column chromatography (silica gel, EtOAc-MeOH 9:1) to afford the title compound (230 mg, 91%). Mp 114–116 °C (CH_2_Cl_2_). ^1^H NMR (400 MHz, CDCl_3_) δ 12.38 (brs, 1H, D_2_O exch., N*H*), 8.52 (d, *J =* 7.85 Hz, 1H, H-3), 8.21 (d, *J =* 8.53 Hz, 1H, H-4’), 8.00 (d, *J =* 7.51 Hz, 1H, H-6), 7.46 (t, *J =* 7.51 Hz, 1H, H-4), 7.08 (t, *J =* 7.51 Hz, 1H, H-5), 6.05 (brs, 1H, D_2_O exch., N*H*CH_2_CH_2_), 5.96 (d, *J =* 8.53 Hz, 1H, H-5’), 3.94 (s, 3H, COOC*H_3_*), 3.43 (m, 2H, NHC*H_2_*CH_2_), 2.61 (t, *J =* 5.80 Hz, 2H, NHCH_2_C*H_2_*), 2.51 (q, *J =* 6.83 Hz, 4H, N(C*H_2_*CH_3_)_2_), 0.97 (t, *J =* 6.83 Hz, 6H, N(CH_2_C*H_3_*)_2_). ^13^C NMR (50 MHz, CDCl_3_) δ 167.3 (CO), 159.6 (C-6’), 151.0 (C-1′), 140.0 (C-4’), 136.3 (C-2), 132.3 (C-4), 131.2 (C-6), 123.7 (C-3), 122.6 (C-5), 120.7 (C-3′), 119.7 (C-1), 102.1 (C-5′), 52.3 (COO*C*H_3_), 51.1 (NHCH_2_*C*H_2_), 46.5 (N(*C*H_2_CH_3_)_2_), 39.1 (NH*C*H_2_CH_2_), 11.7 (N(CH_2_*C*H_3_)_2_). HR-MS (ESI) *m*/*z*: calcd. for C_19_H_26_N_2_O_4_^+^: [M1 + H]^+^ = 388.1979, found 388.1987.

*Methyl 2-((6-((2-(dimethylamino)ethyl)amino)-3-nitropyridin-2-yl)amino)benzoate* (**7**): This compound was prepared by an analogous procedure as described for the preparation of **6** using compound **4** and *N*,*N*-dimethylethylenediamine. Yield: 79 %. Mp 122–124 °C (CH_2_Cl_2_). ^1^H NMR (400 MHz, CDCl_3_) δ 12.37 (brs, 1H, D_2_O exch., N*H*), 8.52 (d, *J* = 7.85 Hz, 1H, H-3), 8.24 (d, *J* = 8.53 Hz, 1H, H-4’), 8.02 (d, *J* = 7.85 Hz, 1H, H-6), 7.49 (t, *J* =7.85 Hz, 1H, H-4), 7.10 (t, *J* = 7.85 Hz, 1H, H-5), 5.98 (m, 2H, N*H*CH_2_CH_2_, D_2_O exch., H-5’), 3.96 (s, 3H, COOC*H_3_*), 3.46 (m, 2H, NHC*H_2_*CH_2_), 2.52 (t, *J* = 5.80 Hz, 2H, NHCH_2_C*H_2_*), 2.25 (s, 6H, N(C*H_3_*)_2_). ^13^C NMR (50 MHz, CDCl_3_) δ 167.3 (CO), 159.6 (C-6’), 150.9 (C-2’), 140.0 (C-4’), 136.4 (C-2), 132.3 (C-4), 131.1 (C-6), 123.7 (C-3), 122.6 (C-5), 120.8 (C-3′), 119.7 (C-1), 101.8 (C-6’), 57.4 (NHCH_2_*C*H_2_), 52.3 (COO*C*H_3_), 45.0 (N(*C*H_3_)_2_)), 39.0 (NH*C*H_2_CH_2_). HR-MS (ESI) *m*/*z*: calcd. for C_17_H_22_N_5_O_4_^+^: [M1 + H]^+^ = 360.1666, found 360.1672.

*Methyl 2-((6-((3-(diethylamino)propyl)amino)-3-nitropyridin-2-yl)amino)benzoate* (**8**): This compound was prepared by an analogous procedure as described for the preparation of **6** using compound **4** and *N*,*N*-diethyl-1,3-propanediamine. Yield: 80%. Mp 116–117 °C (CH_2_Cl_2_). ^1^H NMR (400 MHz, CDCl_3_) δ 12.38 (brs, 1H, D_2_O exch., N*H*), 8.61 (d, *J* = 8.87 Hz, 1H, H-3), 8.18 (d, *J* = 8.77 Hz, 1H, H-4’), 8.01 (d, *J* = 7.60 Hz, 1H, H-6), 7.97 (brs, 1H, D_2_O exch., N*H*CH_2_ CH_2_CH_2_), 7.46 (t, *J* = 7.60 Hz, 1H, H-4), 7.08 (t, *J* = 7.60 Hz, 1H, H-5), 5.87 (d, *J* = 8.7 Hz, 1H, H-5’), 3.95 (s, 3H, COOC*H_3_*), 3.51 (m, 2H, NHC*H_2_*CH_2_CH_2_), 2.63 (t, *J* = 5.55 Hz, 2H, NHCH_2_CH_2_C*H_2_*), 2.52 (q, *J* = 7.89 Hz, 4H, N(C*H_2_*CH_3_)_2_), 1.73 (quintet, *J* = 5.55 Hz, 2H, NHCH_2_C*H_2_*CH_2_), 1.03 (t, *J* =7.89 Hz, 6H, N(CH_2_C*H_3_*)_2_). ^13^C NMR (50 MHz, CDCl_3_) δ 167.3 (CO), 159.7 (C-6’), 151.2 (C-2’), 140.2 (C-4’), 136.0 (C-2), 132.3 (C-4), 131.1 (C-6), 123.8 (C-3), 122.4 (C-5), 120.4 (C-3’), 119.4 (C-1), 102.2 (C-5’) 52.7 (COO*C*H_3_), 52.2 (NHCH_2_CH_2_*C*H_2_), 46.7 (N(*C*H_2_CH_3_)_2_), 42.6 (NH*C*H_2_CH_2_CH_2_), 24.9 (NHCH_2_*C*H_2_CH_2_), 11.7 (N(CH_2_*C*H_3_)_2_). HR-MS (ESI) *m*/*z*: calcd. for C_20_H_28_N_5_O_4_^+^: [M1 + H]^+^ = 402.2136, found 402.2133.

*Methyl 2-((6-((3-(dimethylamino)propyl)amino)-3-nitropyridin-2-yl)amino)benzoate* (**9**): This compound was prepared by an analogous procedure as described for the preparation of **6** using compound **4** and *N*,*N*-dimethyl-1,3-propanediamine. Yield: 90%. Mp 115–117 °C (CH_2_Cl_2_). ^1^H NMR (400 MHz, CDCl_3_) δ 12.43 (brs, 1H, D_2_O exch., N*H*), 8.58 (d, *J* = 8.62 Hz, 1H, H-3), 8.17 (brs, 1H, H-4’), 8.00 (d, *J* = 8.62 Hz, 1H, H-6), 7.46 (t, *J* = 8.62 Hz, 1H, H-4), 7.24 (brs, 1H, D_2_O exch., N*H*CH_2_CH_2_CH_2_), 7.08 (t, *J* = 8.62 Hz, 1H, H-5), 5.93 (d, *J* = 8.62 Hz, 1H, H-5’), 3.94 (s, 3H, COOC*H_3_*), 3.49 (m, 2H, NHC*H_2_*CH_2_CH_2_), 2.38 (t, *J* = 6.74 Hz, 2H, NCH_2_CH_2_C*H_2_*), 2.21 (s, 6H, N(C*H_3_*)_2_)), 1.73 (quintet, *J* = 6.74 Hz, 2H, NHCH_2_C*H_2_*CH_2_). ^13^C NMR (50 MHz, CDCl_3_) δ 167.3 (CO), 159.9 (C-6’), 151.1 (C-2’), 140.2 (C-4’), 135.8 (C-2), 132.3 (C-4), 131.1 (C-6), 123.8 (C-3), 122.5 (C-5), 120.3 (C-3’), 119.4 (C-1), 102.6 (C-5’), 58.5 (NHCH_2_CH_2_*C*H_2_), 52.3 (COO*C*H_3_), 45.2 (N(*C*H_3_)_2_), 41.8 (NH*C*H_2_CH_2_CH_2_), 25.9 NH(CH_2_*C*H_2_CH_2_). HR-MS (ESI) *m*/*z*: calcd. for C_18_H_24_N_5_O_4_^+^: [M1 + H]^+^ = 374.1823, found 374.1818.

*Methyl 2-((6-((2-(diethylamino)ethyl)amino)-3-nitropyridin-2-yl)amino)-5-methoxybenzoate* (**10**): This compound was prepared by an analogous procedure as described for the preparation of **6** using compound **5** and *N*,*N*-diethylethylenediamine. Yield: 97 %. Mp 153–155 °C (CH_2_Cl_2_). ^1^H NMR (400 MHz, CDCl_3_) δ 12.12 (brs, 1H, D_2_O exch., N*H*), 8.34 (d, *J* = 9.33 Hz, 1H, H-3), 8.23 (d, *J* = 8.87 Hz, 1H, H-4’), 7.50 (d, *J* = 3.11 Hz, 1H, H-6), 7.06 (dd, *J* = 9.33, 3.11 Hz, 1H, H-4), 5.93 (m, 2H, D_2_O exch, N*H*CH_2_CH_2_, H-5’), 3.95 (s, 3H, COOC*H_3_*), 3.85 (s, 3H, OC*H_3_*), 3.40 (m, 2H, NHC*H_2_*CH_2_), 2.46–2.66 (m, 6H, NHCH_2_C*H_2_*_,_ N(C*H_2_*CH_3_)_2_), 1.00 (t, *J* = 7.23 Hz, 6H, N(CH_2_C*H_3_*)_2_). ^13^C NMR (50 MHz, CDCl_3_) δ 167.0 (CO), 159.7 (C-6’), 154.9 (C-5), 151.2 (C-2’), 136.3 (C-4’), 133.1 (C-2), 125.9 (C-3), 121.3 (C-2’), 120.1 (C-4), 119.3 (C-1), 114.1 (C-6), 101.9 (C-5’), 55.6 (O*C*H_3_), 52.4 (COO*C*H_3_), 51.1 (NHCH_2_*C*H_2_), 46.5 (N(*C*H_2_CH_3_)_2_), 39.0 (NH*C*H_2_CH_2_), 11.6 (N(CH_2_*C*H_3_)_2_). HR-MS (ESI) *m*/*z*: calcd. for C_20_H_28_N_5_O_5_^+^: [M1 + H]^+^ = 418.2085, found 418.2089.

*Methyl 2-((6-((2-(dimethylamino)ethyl)amino)-3-nitropyridin-2-yl)amino)-5-methoxybenzoate* (**11**): This compound was prepared by an analogous procedure as described for the preparation of **6** using compound **5** and *N*,*N*-dimethylethylenediamine. Yield: 94 %. Mp 158–160 °C (CH_2_Cl_2_). ^1^H NMR (400 MHz, CDCl_3_) δ 12.11 (brs, 1H, D_2_O exch., N*H*), 8.33 (d, *J* = 9.22 Hz, 1H, H-3), 8.20 (d, *J* = 9.22 Hz, 1H, H-4’), 7.50 (d, *J* = 3.07 Hz, 1H, H-6), 7.06 (dd, *J* = 9.22, 3.07 Hz, 1H, H-4), 5.92 (m, 2H, D_2_O exch., N*H*CH_2_CH_2_, H-5’), 3.94 (s, 3H, COOC*H_3_*), 3.84 (s, 3H, OC*H_3_*), 3.42 (m, 2H, NHC*H_2_*CH_2_), 2.48 (t, *J* = 5.80 Hz, 2H, NHCH_2_C*H_2_*), 2.23 (s, 6H, N(C*H_3_*)_2_)_._^13^C NMR (50 MHz, CDCl_3_) δ 166.9 (CO), 159.7 (C-6’), 154.9 (C-5), 151.1 (C-2’), 136.3 (C-4’), 133.0 (C-2’), 131.1 (C-2), 125.9 (C-3), 121.2 (C-3’), 120.2 (C-4), 119.3 (C-1), 114.2 (C-6), 102.3 (C-5’), 57.4 (O*C*H_3_), 55.5 (COO*C*H_3_), 52.3 (NHCH_2_*C*H_2_), 44.9 (N(*C*H_3_)_2_), 38.9 (NH*C*H_2_CH_2_). HR-MS (ESI) *m*/*z*: calcd. for C_18_H_24_N_5_O_5_^+^: [M1 + H]^+^ = 390.1772, found 390.1766.

*Methyl 2-((6-((3-(diethylamino)propyl)amino)-3-nitropyridin-2-yl)amino)-5-methoxybenzoate* (**12**): This compound was prepared by an analogous procedure as described for the preparation of **6** using compound **5** and *N*,*N*-diethyl-1,3-propanediamine. Yield: 89 %. Mp 140–142 °C (CH_2_Cl_2_). ^1^H NMR (400 MHz, CDCl_3_) δ 12.18 (brs, 1H, D_2_O exch., N*H*), 8.40 (d, *J* = 9.39 Hz, 1H, H-3), 8.18 (brs, 1H, H-4’), 7.96 (brs, 1H, D_2_O exch., N*H*CH_2_CH_2_CH_2_,), 7.50 (d, *J* = 2.47 Hz, 1H, H-6), 7.05 (dd, *J* = 9.22, 3.07 Hz, 1H, H-4), 5.83 (d, *J* = 8.61 Hz, 1H, H-5’), 3.95 (s, 3H, COOC*H_3_*), 3.84 (s, 3H, OC*H_3_*), 3.49 (m, 2H, NHC*H_2_*CH_2_CH_2_,), 2.50–2.59 (m, 6H, NHCH_2_CH_2_C*H_2_*, and N(C*H_2_*CH_3_)_2_), 1.72 (quintet, 2H, NHCH_2_C*H_2_*CH_2_), 1.04 (t, *J* = 7.04 Hz, 6H, N(CH_2_C*H_3_*)_2)._^13^C NMR (50 MHz, CDCl_3_) δ 167.0 (CO), 159.8 (C-6’), 154.9 (C-5), 151.5 (C-2’), 136.0 (C-4’), 133.3 (C-2), 126.0 (C-3), 121.2 (C-3’), 119.3 (C-1, C-4), 114.1 (C-6), 102.5 (C-5’), 55.6 (O*C*H_3_), 52.9 (COO*C*H_3_), 52.4 (NHCH_2_CH_2_*C*H_2_,), 46.9 (N(*C*H_2_CH_3_)_2_), 42.6 (NH*C*H_2_CH_2_CH_2_), 25.0 (NHCH_2_*C*H_2_CH_2_), 11.6 (N(CH_2_*C*H_3_)_2_)). HR-MS (ESI) *m*/*z*: calcd. for C_21_H_30_N_5_O_5_^+^: [M1 + H]^+^ = 432.2241, found 432.2245.

*Methyl 2-((6-((3-(dimethylamino)propyl)amino)-3-nitropyridin-2-yl)amino)-5-methoxybenzoate* (**13**): This compound was prepared by an analogous procedure as described for the preparation of **6** using compound **5** and *N*,*N*-dimethyl-1,3-propanediamine. Yield: 95%. Mp 163–164 °C (CH_2_Cl_2_). ^1^H NMR (400 MHz, CDCl_3_) δ 12.15 (brs, 1H, D_2_O exch., N*H*), 8.37 (d, *J* = 9.23 Hz, 1H, H-3), 8.18 (brs, 1H, H-4’), 7.49 (d, *J* = 3.07 Hz, 1H, H-6), 7.16 (brs, 1H, D_2_O exch., N*H*CH_2_CH_2_CH_2_), 7.05 (dd, *J* = 9.22, 3.07 Hz, 1H, H-4), 5.86 (d, *J* = 8.87 Hz, 1H, H-5’), 3.94 (s, 3H, COOC*H_3_*), 3.83 (s, 3H, OC*H_3_*), 3.47 (m, 2H, NHC*H_2_*CH_2_CH_2_), 2.39 (t, *J* = 6.14 Hz, 2H, NHCH_2_CH_2_C*H_2_*), 2.22 (s, 6H, N(C*H_3_*)_2_), 1.71 (m, 2H, NHCH_2_C*H_2_*CH_2_). ^13^C NMR (50 MHz, CDCl_3_) δ 167.0 (CO), 159.9 (C-6’), 154.9 (C-5), 151.3 (C-2’), 136.1 (C-4’), 133.1 (C-2), 126.0 (C-3), 121.2 (C-3’), 119.8 (C-1), 119.3 (C-4), 114.1 (C-6), 101.9 (C-6’), 60.3 (NHCH_2_CH_2_*C*H_2_), 55.6 (O*C*H_3_), 52.4 (COO*C*H_3_), 45.4 (N(*C*H_3_)_2_)), 41.9 (NH*C*H_2_CH_2_CH_2_), 25.7 (NHCH_2_*C*H_2_CH_2_). HR-MS (ESI) *m*/*z*: calcd. for C_19_H_26_N_5_O_5_^+^: [M1 + H]^+^ = 404.1928, found 404.1923.

*9-((2-(Diethylamino)ethyl)amino)-6-nitro-11H-pyrido[2,1-b]quinazolin-11-one* (**22**): To a solution of ester **6** (160 mg, 0.413 mmol) in methanol (20 mL) was added dropwise, at room temperature, a cold 20% NaOH solution (1 mL). The mixture was stirred for 14 h at room temperature and then poured into water and neutralized (pH 7) with a 4 % HCl solution. The resulting carboxylic acid 14 was filtered, dried over P_2_O_5_ and then, dissolved in a 1:2 *v/v* mixture of trifluoroacetic anhydride and trifluoroacetic acid (1.5 mL). The resulting mixture was heated at 60 °C for 6 h and, upon cooling, it was poured into ice-water and neutralized with solid sodium carbonate. The precipitate was filtered and air-dried to give crude **22**, which was purified by column chromatography (silica gel) using a mixture of CH_2_Cl_2_-MeOH 10:1, as the eluent, to provide the title compound as a yellow solid (110 mg, 75%). Mp 100–101°C (EtOAc). ^1^H NMR (400 MHz, CDCl_3_) δ 11.31 (brs, 1H, D_2_O exch., N*H*), 8.32 (d, *J* = 9.39 Hz, 1H, H-7), 8.25 (d, *J* = 8.22 Hz, 1H, H-1), 7.79 (t, *J* = 8.22 Hz, 1H, H-3), 7.70 (d, *J*= 8.22 Hz, 1H, H-4), 7.38 (t, *J* = 8.22 Hz, 1H, H-2), 5.58 (d, *J* = 9.39 Hz, 1H, H-8), 3.37 (m, 2H, NHC*H_2_*CH_2_), 2.38 (m, 2H, NHCH_2_C*H_2_*), 2.66 (m, 4H, N(C*H_2_*CH_3_)_2_), 1.12 (t, *J* = 6.93 Hz, 6H, N(CH_2_C*H_3_*)_2_). ^13^C NMR (50 MHz, CDCl_3_) δ 165.5 (CO), 154.8 (C-5a), 146.8 (C-4a), 141.7 (C-9), 137.5 (C-7), 136.0 (C-3), 128.2 (C-6), 127.3 (C-1), 127.0 (C-4), 125.4 (C-2), 117.3 (C-1), 86.7 (C-8), 49.9 (NCH_2_*C*H_2_), 46.6 (N(*C*H_2_CH_3_)_2_), 42.0 (NH*C*H_2_CH_2_), 11.7 (N(CH_2_*C*H_3_)_2_). HR-MS (ESI) *m*/*z*: calcd. for C_18_H_22_N_5_O_3_^+^: [M1 + H]^+^ = 356.1717, found 356.1725.

*9-((2-(Dimethylamino)ethyl)amino)-6-nitro-11H-pyrido[2,1-b]quinazolin-11-one* (**23**): This compound was prepared by an analogous procedure as described for the preparation of **22** using compound **7.** Yield: 71 %. Mp 127–129 °C (EtOAc). ^1^H NMR (400 MHz, CDCl_3_) δ 11.28 (brs, 1H, D_2_O exch., NH), 8.29 (d, *J* = 8.88 Hz, 1H, H-7), 8.22 (d, *J* = 7.85 Hz, 1H, H-1), 7.77 (t, *J* = 7.85 Hz, 1H, H-3), 7.67 (d, *J* = 7.85 Hz, 1H, H-4), 7.36 (t, *J* =7.85 Hz, 1H, H-2), 5.54 (d, *J* = 8.88 Hz, 1H, H-8), 3.35 (m, 2H, NHC*H_2_*CH_2_), 2.67 (t, *J* = 6.14 Hz, 2H, NHCH_2_C*H_2_*), 2.35 (s, 6H, (N(C*H_3_*)_2_). ^13^C NMR (50 MHz, CDCl_3_) δ 165.7 (CO), 154.8 (C-5a), 146.8 (C-4a), 141.6 (C-9), 137.5 (C-7), 136.1 (C-3), 128.5 (C-6), 127.3 (C-1), 127.0 (C-4), 125.6 (C-2), 117.2 (C-11a), 86.5 (C-8), 56.4 (NHCH_2_*C*H_2_), 45.1 (N(*C*H_3_)_2_), 41.6 (NH*C*H_2_CH_2_). HR-MS (ESI) *m*/*z*: calcd. for C_18_H_22_N_5_O_3_^+^: [M1 + H]^+^ = 328.1404, found 328.1410.

*9-((3-(Diethylamino)propyl)amino)-6-nitro-11H-pyrido[2,1-b]quinazolin-11-one* (**24**): This compound was prepared by an analogous procedure as described for the preparation of **22** using compound **8**. Yield: 73 %. Mp 110–111 °C (EtOAc). ^1^H NMR (400 MHz, CDCl_3_) δ 11.30 (brs, 1H, D_2_O exch., NH), 8.31 (d, *J* = 9.21 Hz, 1H, H-7), 8.24 (d, *J* = 8.09 Hz, 1H, H-1), 7.78 (t, *J* = 8.32 Hz, 1H, H-3), 7.70 (d, *J* = 8.30 Hz, 1H, H-4), 7.37 (t, *J* = 7.11 Hz, 1H, H-2), 5.56 (d, *J* = Hz, 1H, H-8), 3.34 (q, *J* = 6.7 Hz, 3H, NHC*H_2_*CH_2_CH_2_), 2.81 (t, *J* = 6.5 Hz, 2H, NHCH_2_CH_2_CH*_2_*), 2.64 (q, 4H, N(CH_2_CH_3_)_2_), 1.58 (quintet, *J* = 6.6 Hz, 2H, NHCH_2_C*H_2_*CH_2_), 1.11 (t, 6H, N(CH_2_C*H_3_*)_2_). ^13^C NMR (50 MHz, CDCl_3_) δ 165.7 (CO), 154.8 (C-5a), 146.8 (C-4a), 141.5 (C-9), 137.3 (C-7), 136.0 (C-3), 128.2 (C-6), 127.0 (C-1), 126.9 (C-4), 125.5 (C-2), 117.1 (C-11a), 86.5 (C-8), 50.3 (NHCH_2_CH_2_*C*H_2_), 46.7 (N(*C*H_2_CH_3_)_2_), 42.7 (NH*C*H_2_CH_2_CH_2_), 25.6 (NHCH_2_*C*H_2_CH_2_), 11.3 (N(CH_2_*C*H_3_)_2_. HR-MS (ESI) *m*/*z*: calcd. for C_19_H_24_N_5_O_3_^+^: [M1 + H]^+^ = 370.1874, found 370.1867.

*9-((3-(Dimethylamino)propyl)amino)-6-nitro-11H-pyrido[2,1-b]quinazolin-11-one* (**25**): This compound was prepared by an analogous procedure as described for the preparation of **22** using compound **9**. Yield: 74 %. Mp 106–107 °C (EtOAc). ^1^H NMR (400 MHz, CDCl_3_) δ 11.34 (brs, 1H, D_2_O exch., N*H*), 8.28 (d, *J* = 9.2 Hz, 1H, H-7), 8.18 (d, *J* = 8.1 Hz, 1H, H-1), 7.77 (t, *J* = 7.1 Hz, 1H, H-3), 7.69 (d, *J* = 8.2 Hz, 1H, H-4), 7.36 (t, *J* = 7.9 Hz, 1H, H-2), 5.61 (d, *J* = 9.3 Hz, 1H, H-8), 3.39 (q, *J* = 6.7 Hz, 2H, NHC*H_2_*CH_2_CH_2_), 2.48 (t, *J* = 6.5 Hz, 2H, NHCH_2_CH_2_CH*_2_*), 2.29 (s, 6H, N(CH*_3_*)_2_), 1.92 (quintet, *J*= 6.6 Hz, 2H, NHCH_2_C*H_2_*CH_2_). ^13^C NMR (50 MHz, CDCl_3_) δ 166.0 (CO), 155.0 (C-5a), 146.9 (C-4a), 141.6 (C-9), 137.4 (C-7), 136.2 (C-3), 128.4 (C-6), 127.1 (C-1, C-4), 125.6 (C-2), 117.1 (C-11a), 86.5 (C-8), 56.8 (NHCH_2_CH_2_*C*H_2_), 45.3 (N(*C*H_3_)_2_), 42.5 (NH*C*H_2_CH_2_CH_2_), 25.7 (NHCH_2_*C*H_2_CH_2_). HR-MS (ESI) *m*/*z*: calcd. for C_17_H_20_N_5_O_3_^+^: [M1 + H]^+^ = 342.1561, found 342.1555.

*9-((2-(Diethylamino)ethyl)amino)-2-methoxy-6-nitro-11H-pyrido[2,1-b]quinazolin-11-one* (**26**): This compound was prepared by an analogous procedure as described for the preparation of **22** using compound **10**. Yield: 71 %. Mp 105–107 °C (EtOAc). ^1^H NMR (400 MHz, CDCl_3_) δ 11.24 (brs, 1H, D_2_O exch., NH), 8.19 (d, *J* = 9.22 Hz, 1H, H-7), 7.53 (d, *J* = 8.87 Hz, 1H, H-4), 7.34 (d, *J* = 2.73 Hz, 1H, H-1), 7.30 (dd, *J* = 8.87, 2.73 Hz, 1H, H-3), 5.44 (d, *J* = 9.22 Hz, 1H, H-8), 3.83 (s, 3H, OC*H_3_*), 3.29 (t, *J* = 6.14 Hz, 2H, NHC*H_2_*CH_2_), 2.77 (t, *J* = 6.14 Hz, 2H, NHCH_2_C*H_2_*), 2.62 (q, *J* = 7.1 Hz, 4H, N(C*H_2_*CH_3_)_2_), 1.08 (t, *J* = 7.17 Hz, 6H, N(CH_2_C*H_3_*)_2_). ^13^C NMR (50 MHz, CDCl_3_) δ 164.7 (CO), 157.3 (C-2), 154.5 (C-5a), 141.4 (C-4a), 139.7 (C-9), 136.8 (C-7), 128.5 (C-4), 127.7 (C-6), 127.0 (C-3), 117.6 (C-11a), 105.3 (C-1), 86.6 (C-8), 55.6 (O*C*H_3_), 49.8 (NHCH_2_*C*H_2_), 46.5 (N(C*H*_2_*C*H_3_)_2_), 41.9 (NH*C*H_2_CH_2_), 11.6 (N(C*H*_2_*C*H_3_)_2_). HR-MS (ESI) *m*/*z*: calcd. for C_19_H_24_N_5_O_4_^+^: [M1 + H]^+^ = 386.1823, found 386.1826.

*9-((2-(Dimethylamino)ethyl)amino)-2-methoxy-6-nitro-11H-pyrido[2,1-b]quinazolin-11-one* (**27**): This compound was prepared by an analogous procedure as described for the preparation of **22** using compound **11**. Yield: 79 %. Mp 118–120 °C (EtOAc). ^1^H NMR (400 MHz, CDCl_3_) δ 11.24 (brs, 1H, D_2_O exch., N*H*), 8.26 (d, *J* = 9.39 Hz, 1H, H-7), 7.64 (d, *J* = 8.61 Hz, 1H, H-4), 7.52 (d, *J* = 2.74 Hz, 1H, H-1), 7.40 (dd, *J* = 8.61, 2.47 Hz, 1H, H-3), 5.51 (d, *J* = 9.39 Hz, 1H, H-8), 3.90 (3H, s, OC*H_3_*), 3.36 (m, 2H, NHC*H_2_*CH_2_), 2.69 (t, *J* = 5.48 Hz, 2H, NHCH_2_C*H_2_*), 2.37 (s, 6H, N(C*H_3_*)_2_). ^13^C NMR (50 MHz, CDCl_3_) δ 165.2 (CO), 157.6 (C-2), 154.6 (C-5a), 141.7 (C-4a), 139.9 (C-9), 136.7 (C-7), 128.7 (C-4), 128.5 (C-6), 127.4 (C-3), 117.7 (C-11a), 105.4 (C-1), 86.2 (C-8), 56.4 (NHCH_2_*C*H_2_), 55.8 (O*C*H_3_), 45.1 (N(*C*H_3_)_2_), 41.6 (NH*C*H_2_CH_2_). HR-MS (ESI) *m*/*z*: calcd. for C_17_H_20_N_5_O_4_^+^: [M1 + H]^+^ = 358.1510, found 358.1517.

*9-((3-(Diethylamino)propyl)amino)-2-methoxy-6-nitro-11H-pyrido[2,1-b]quinazolin-11-one* (**28**): This compound was prepared by an analogous procedure as described for the preparation of **22** using compound **12**. Yield: 80 %. Mp 113–114 °C (EtOAc). ^1^H NMR (400 MHz, CDCl_3_) δ 11.30 (brs, 1H, D_2_O exch., N*H*), 8.26 (d, *J* = 9.39 Hz, 1H, H-7), 7.65 (d, *J* = 9.00 Hz, 1H, H-4), 7.48 (d, *J* = 2.47 Hz, 1H, H-1), 7.41 (dd, *J* = 9.00, 2.74 Hz, 1H, H-3), 5.58 (d, *J* = 9.39 Hz, 1H, H-8), 3.90 (s, 3H, OC*H_3_*), 3.38 (m, 2H, NHC*H_2_*CH_2_CH_2_), 2.59 (m, 6H, NHCH_2_CH_2_C*H_2_* and N(C*H_2_*CH_3_)_2_), 1.91 (quintet, 2H, NHCH_2_C*H_2_*CH_2_), 1.05 (t, *J* = 7.11 Hz, 6H, N(CH_2_C*H_3_*)_2_). ^13^C NMR (50 MHz, CDCl_3_) δ 165.4 (CO), 157.7 (C-2), 154.7 (C-5a), 141.9 (C-4a), 139.9 (C-9), 136.6 (C-7), 128.9 (C-4), 128.6 (C-6), 127.4 (C-3), 117.7 (C-11a), 105.3 (C-1), 86.1 (C-8), 55.7 (O*C*H_3_), 50.2 (NHCH_2_CH_2_*C*H_2_), 46.8 (N(*C*H_2_CH_3_)_2_), 42.5 (NH*C*H_2_CH_2_CH_2_), 25.9 (NHCH_2_*C*H_2_CH_2_), 11.5 (N(CH_2_*C*H_3_)_2_). HR-MS (ESI) *m*/*z*: calcd. for C_20_H_26_N_5_O_4_^+^: [M1 + H]^+^ = 400.1979, found 400.1984.

*9-((3-(Dimethylamino)propyl)amino)-2-methoxy-6-nitro-11H-pyrido[2,1-b]quinazolin-11-one* (**29**): This compound was prepared by an analogous procedure as described for the preparation of **22** using compound **13**. Yield: 71%. Mp 130–131 °C (EtOAc). ^1^H NMR (400 MHz, CDCl_3_) δ 11.31 (brs, 1H, D_2_O exch., N*H*), 8.23 (d, *J* = 9.2 Hz, 1H, H-7), 7.61 (d, *J* = 8.9 Hz, 1H, H-4), 7.44 (d, *J* = 2.6 Hz, 1H, H-1), 7.38 (dd, *J* = 8.9, 2.62 Hz, 1H, H-3), 5.55 (d, *J* = 9.2 Hz, 1H, H-8), 3.88 (s, 3H, OC*H_3_*), 3.37 (m, 2H, NHC*H_2_*CH_2_CH_2_), 2.45 (t, *J* = 6.6 Hz, 2H, NHCH_2_CH_2_C*H_2_*), 2.27 (s, 6H, (N(C*H_3_*)_2_), 1.92 (quintet, 2H, *J* = 6.7 Hz, NHCH_2_C*H_2_*CH_2_). ^13^C NMR (50 MHz, CDCl_3_) δ 165.3 (CO), 157.6 (C-2), 154.7 (C-5a), 141.8 (C-4a), 139.9 (C-9), 136.6 (C-7), 128.8 (C-4), 128.4 (C-6), 127.3 (C-3), 117.6 (C-11a), 105.2 (C-1), 86.1 (C-8), 56.7 (NHCH_2_CH_2_*C*H_2_), 55.7 (O*C*H_3_), 45.4 (N(*C*H_3_)_2_), 42.3 (NH*C*H_2_CH_2_CH_2_), 25.9 (NHCH_2_*C*H_2_CH_2_). HR-MS (ESI) *m*/*z*: calcd. for C_18_H_22_N_5_O_4_^+^: [M1 + H]^+^ = 372.1666, found 372.1660.

### 3.3. Biological Assays and Experiments

The cytotoxic activity of the synthetic compounds was tested against the human cancer cell lines HCT-116 (colorectal), MES-SA (uterine sarcoma) and its variant MES-SA/Dx5, which is 100 times more resistant to Dx. HCT-116 was purchased from the American Tissue Culture Collection (ATCC, Manassas, VA, USA); MES-SA and MES-SA/Dx5 were kindly donated by Doctors D. Kletsas and H. Pratsinis (Institute of Biosciences and Applications, NCSR “Demokritos”, Athens, Greece). Cells were grown as monolayer in culture medium RPMI-1640, supplemented with 10% heat-inactivated fetal bovine serum, 10 mM Hepes, 10 U/mL penicillin, 10 μg/mL streptomycin and 5 mg/mL gentamycin (all from Lonza, Cologne, Germany) at 37 °C in a humidified 5% CO2 incubator and passaged by trypsinization every 2 to 3 days.

Stock concentrations of the compounds (10.0 mg/mL) were prepared in DMSO (Sigma-Aldrich, Darmstadt, Germany) and stored at −20 °C. Cytotoxicity was evaluated by the MTT reduction assay [26], allowing to estimate the number of metabolically active/viable cells present in culture. Briefly, cells were seeded in 96-well plates (Greiner Bio-One GmbH, Frickenhausen, Germany; 5 × 10^3^ cells/well) and allowed to adhere overnight. On the day of the experiment, stock solutions were serially diluted in culture medium (200.0 to 6.25 μM), added to the plates and incubated at 37 °C for 72 h. Cells incubated in culture medium or in medium containing the equivalent amount of DMSO, and cells incubated in the presence of Dx or Mitox (Sigma-Aldrich) were used as negative and positive controls, respectively. The MTT reagent (Sigma-Aldrich; 1 mg/mL in phosphate buffered saline; 100 μL/well) was added to each well during the last 4 h of incubation. The formazan crystals formed were dissolved by adding 0.1 M HCl in 2-propanol (Sigma-Aldrich; 100 μL/well) and absorption was measured using an ELISA reader (Denley WeScan, Finland) at 545 nm with the reference filter set at 690 nm. All cultures were set in triplicate. The IC_50_ values were calculated by plotting the sigmoidal curves (response vs. log concentration) fitted to the 4-parameter logistic equation using non-linear regression (GraphPad Prism version 6.0 for Windows from GraphPad Software, Inc., La Jolla, CA, USA).

### 3.4. Computational Chemistry

In order to explain the difference of the derivatives’ hydrolysis rates, a series of electronic property calculations were carried out. All electronic structure calculations were performed with the Gaussian09 computational chemistry package [27]. The drawn structure was initially optimized at the semi-empirical theory employing the PM3 Hamiltonian and at the final geometry employing DFT calculation with B3LYP 6-31G basis set. A population analysis was performed by the NBO 3 program as embedded in the Gaussion software, writing the fchk files as well as the .47 file. The estimation of the Fukui functions as well as the philicities were achieved using the UCA-FUKUI 2.1 program [28]. The finite difference approximation based on BO analysis as well as the FMO approximation based on the NBO coefficients have been used. The electronic properties of the molecule—i.e., the atomic charges—are based on the MK ESP-fitting atomic charge scheme [29], whereas the bond order analysis was based on the Mayer method. Furthermore, the QTAIM theory of Bader et al. was employed in order to investigate the partitioning of the electrostatic density between the atoms as well as to investigate the formation of bonds that are not detected by the classical methodologies [30]. The MutiWFN software [31] was employed for the population, the bond order, and the QTAIM analyses.

### 3.5. Chemical Analysis

The hydrolysis rate of the two products was studied by mass spectrometry employing a Waters QToF premiere (Waters, Manchester, UK). The introduction of the sample was done using the embedded syringe. Solutions of the two molecules were freshly prepared in acetonitrile at the 1 mg/mL level and the samples were diluted in water at the 20 μg/mL level and were directly infused into the instrument. The quasimolecular ions of the hydrolysis products were monitored in the (-) ion mode. The parameters used for the mass spectrometric analysis electrospray voltage at 1.5 kV, sample cone voltage at 35 V, extraction cone voltage at 3 V and the MCP plates were operated at 1850 V. Nitrogen was used both as the desolvation gas and was set at 650 L·h^−1^ and heated at 350 °C. The TOF analyzer was operated in the V optics mode affording a resolution of 9500. The intensity of each ion was monitored over time.

## 4. Conclusions

A series of new aza-acridine analogues bearing a basic side chain have been synthesized and evaluated for their antiproliferative activity. The compounds possessed low biological activity compared to their acridine analogues against three cancer cell lines tested. It was assumed that this could probably be attributed to the hydrolytic instability of the compounds in aqueous media. Thus, a study of two selected analogues—i.e., **25** and **29**—differing by a methoxy group at position 2 of their core, was performed using mass spectrometry and computational chemistry experiments employing the DFT theory. It was proved that the aza-acridines exist in a mixture with their corresponding ring-opened counterparts and the ratio is dependent on the substitution of the aza-acridine core. The combination of approaches followed herein to explain the loss of anticipated activity could lead to the redesign of more stable and thus more potent aza-acridine analogues.

## Figures and Tables

**Table 1:** Inhibition of proliferation (IC_50_ values in μM) of compounds **22**–**29** against HCT-116, MES-SA, and MES-SA/DX5 human tumor cell lines determined after 72 h of exposure.

Compounds	IC_50_ (μM) ^a^			
	HCT-116	MES-SA	MES-SA/Dx5	RF ^b^
**22**	136.03 ± 4.80	72.10 ± 1.90	65.07 ± 3.93	0.90
**23**	114.93 ± 5.99	49.90 ± 3.03	50.30 ± 1.35	1.01
**24**	192.43 ± 10.47	188.10 ± 1.82	164.47 ± 4.20	0.87
**25**	126.30 ± 5.37	51.73 ± 3.47	47.93 ± 1.99	0.93
**26**	15.37 ± 2.34	37.03 ± 3.42	21.27 ± 0.76	0.57
**27**	20.47 ± 3.75	22.27 ± 1.60	10.33 ± 0.55	0.46
**28**	73.13 ± 3.18	47.43 ± 2.56	34.40 ± 1.41	0.73
**29**	67.67 ± 1.39	36.83 ± 1.37	19.47 ± 1.25	0.53
**Dx**	0.183 ± 0.016	0.037 + 0.011	2.680 ± 0.261	72.43
**Mitox**	0.020 ± 0.006	0.007 ± 0.003	0.081± 0.013	11.57

^a^ The results presented are means ± standard deviation (SD) of three independent experiments and are expressed as IC_50_; i.e., the effective concentration reducing viability by 50% compared to unexposed control cells; ^b^ IC_50_ of resistant cells (MES-SA/Dx5)/IC_50_ of sensitive cells (MES-SA).

**Table 2:** Equations of the hydrolysis reactions.

Compound	Equation y = intercept (±Standard Error) + slope (±Standard Error) × t	Correlation Coefficient
**25**	y = 11.49 (±0.001) − 0.065 (±0.0001) × t	0.992
**29**	y = 11.59 (±0.008) − 0.0025 (±0.002) × t	0.994

**Table 3:** Selected geometry characteristics of compounds **25** and **29** describing the lactam as well as its adjacent bonds based on DFT B3LYP 6-31G+. The two molecular structures show only subtle differences.

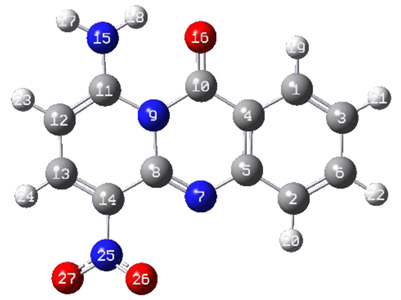				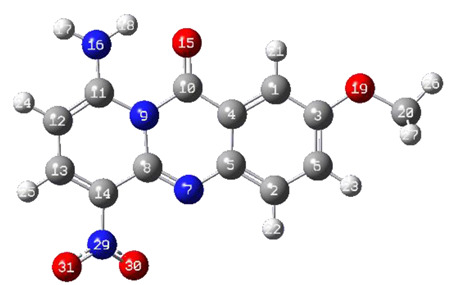			
Compound **25**				Compound **29**			
**Bond Length (Å)**	**9-10**	**4-10**	10-16 *	10-15 *	**8-9**		**4-5**
**25**	1.445	1.449	1.261		1.461		1.415
**29**	1.441	1.448		1.261	1.46493		1.416
**Angle (°)**	**11-9-10**	**9-10-4**	**9-10-16**	**9-10-15**	**4-10-16**	**4-10-15**	**10-4-1**
**25**	121.032	116.810	120.886		122.299		120.034
**29**	120.975	116.838		121.133		122.023	119.779
**Dihedral Angle (°)**	**12-11-9-10**		**11-9-10-16**	**11-9-10-15**	**9-10-4-1**		**10-4-1-3**
**25**	177.74735		2.686		179.446		−178.913
**29**	−177.38246			−2.483	−179.522		179.078
**Atomic Charges (^−^e)**	**N9**	**C10**	**C11**	**C4**	**O16**	**O15**	**C1**
**25**	−0.445	0.761	0.763	−0.374	−0.641		0.004
**29**	−0.522	0.828	0.797	−0.319		−0.635	−0.283

* Numbering is based on the Gaussian 09 numbering. Atom **15** for compound **25** and **16** for compound **29** refer to the carbonyl O.

## Supplementary Materials

The following are available online, Figure S1: ESP electrostatic potential; Figure S2: ELF values; Figure S3: Fukui function of compounds 25 and 29; ^1^H and ^13^C-NMR spectra are available online.
