# Supplementary material for: Synthesis, Biological Evaluation and Stability Studies of Some Novel Aza-Acridine Aminoderivatives
Source: Molecules. 2020 Oct 8;25(19):4584. doi: 10.3390/molecules25194584 (PMC7582268; doi:10.3390/molecules25194584)
Supplement: Supplementary file 1 [file molecules-25-04584-s001.pdf]

molecules

Article

Synthesis, Biological Evaluation and Stability Studies of some Novel Aza-acridine Aminoderivatives.

Maria Karelou [1], Athanasia P. Tragomalou [2], Vasileios Kourafalos [1], Panagiotis Marakos [1], Nicole Pouli [1], Ourania E. Tsitsilonis [2], Evangelos Gikas [3,*], Ioannis K. Kostakis [1,*].

[1] Department of Pharmacy, Division of Pharmaceutical Chemistry, National and Kapodistrian University of Athens, Panepistimiopolis, Zografou, 15771, Athens, Greece.

[2] Section of Animal & Human Physiology, Department of Biology, National & Kapodistrian University of Athens,, Panepistimiopolis, Ilissia, Athens, Greece.

[3] Laboratory of Analytical Chemistry,Department of Chemistry, National and Kapodistrian University of Athens, Panepistimiopolis, Zografou, 157 71, Athens, Greece.

# current address: Division of Endocrinology, Metabolism and Diabetes, 1st Department of Pediatrics, Medical School, "Aghia Sophia" Children's Hospital, National and Kapodistrian University of Athens, Athens, Greece

* Correspondence: vgikas@chem.uoa.gr, ikkostakis@pharm.uoa.gr

Received: date; Accepted: date; Published: date

Contents

Figure S1a and b: Electrostatic potential map ESP of compounds **25** and **29**
Figure S2 a and b: Electron localization function ELF isosurface of compounds **25** and **29**
Figure S3 a and b: Fukui function of compounds **25** and **29**
Figure S4 a, b, c and d: QTAIM of the Rho in the amide plane of compounds **25** and **29** (a and c show the mapped contour c and d the isovalues)
Figure S5: ^{1}H NMR and ^{13}C NMR spectrum of compound **4**.
Figure S6: ^{1}H NMR and ^{13}C NMR spectrum of compound **5**.
Figure S7: ^{1}H NMR and ^{13}C NMR spectrum of compound **6**.
Figure S8: ^{1}H NMR and ^{13}C NMR spectrum of compound **7**.
Figure S9: ^{1}H NMR and ^{13}C NMR spectrum of compound **8**.
Figure S10: ^{1}H NMR and ^{13}C NMR spectrum of compound **9**.
Figure S11: ^{1}H NMR and ^{13}C NMR spectrum of compound **10**.
Figure S12: ^{1}H NMR and ^{13}C NMR spectrum of compound **11**.
Figure S13: ^{1}H NMR and ^{13}C NMR spectrum of compound **12**.
Figure S14: ^{1}H NMR and ^{13}C NMR spectrum of compound **13**.
Figure S15: ^{1}H NMR and ^{13}C NMR spectrum of compound **22**.
Figure S16: ^{1}H NMR and ^{13}C NMR spectrum of compound **23**.
Figure S17: ^{1}H NMR and ^{13}C NMR spectrum of compound **24**.
Figure S18: ^{1}H NMR and ^{13}C NMR spectrum of compound **25**.
Figure S19: ^{1}H NMR and ^{13}C NMR spectrum of compound **26**.

Figure S20: ^{1}H NMR and ^{13}C NMR spectrum of compound **27**.
Figure S21: ^{1}H NMR and ^{13}C NMR spectrum of compound **28**.
Figure S22: ^{1}H NMR and ^{13}C NMR spectrum of compound **29**.
Figure S23: Structures of positive controls.

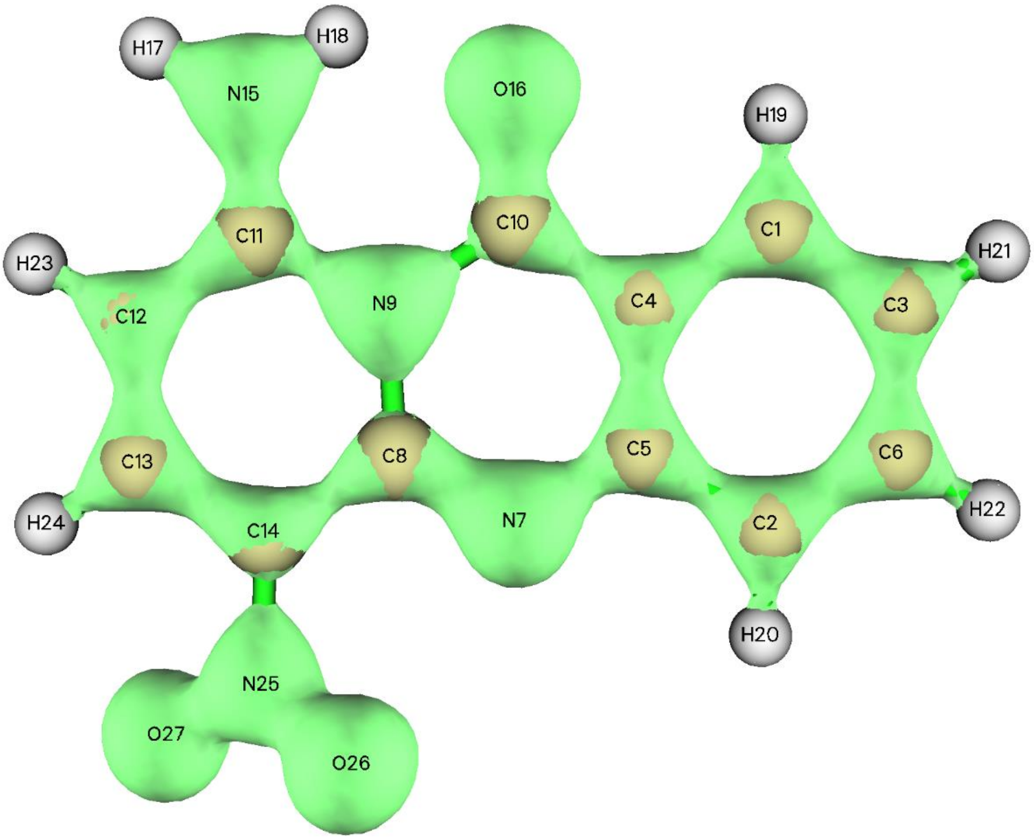

Figure S1a Electrostatic potential map of compound **25**

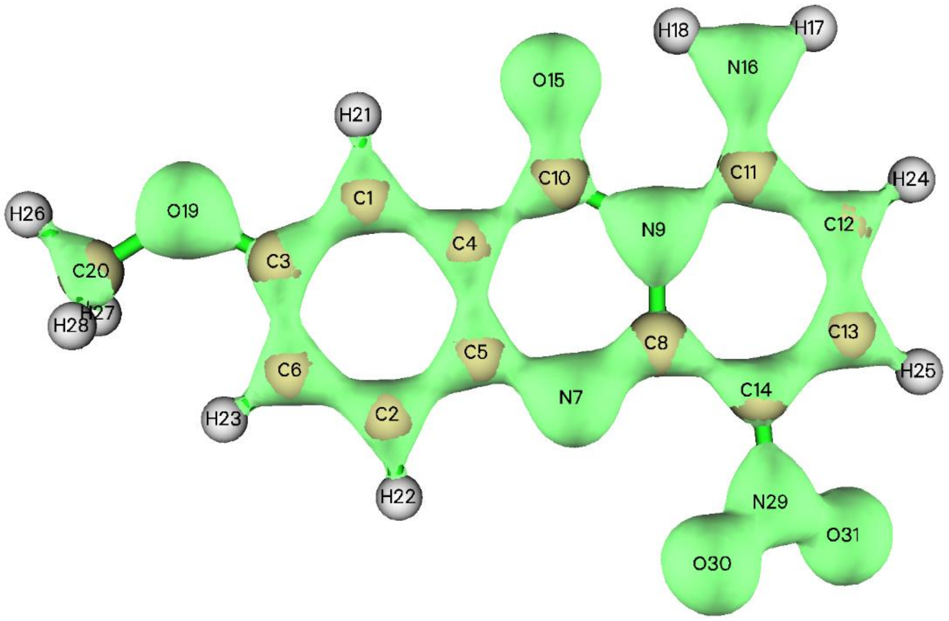

Figure S1b. Electrostatic potential map of compound **29**

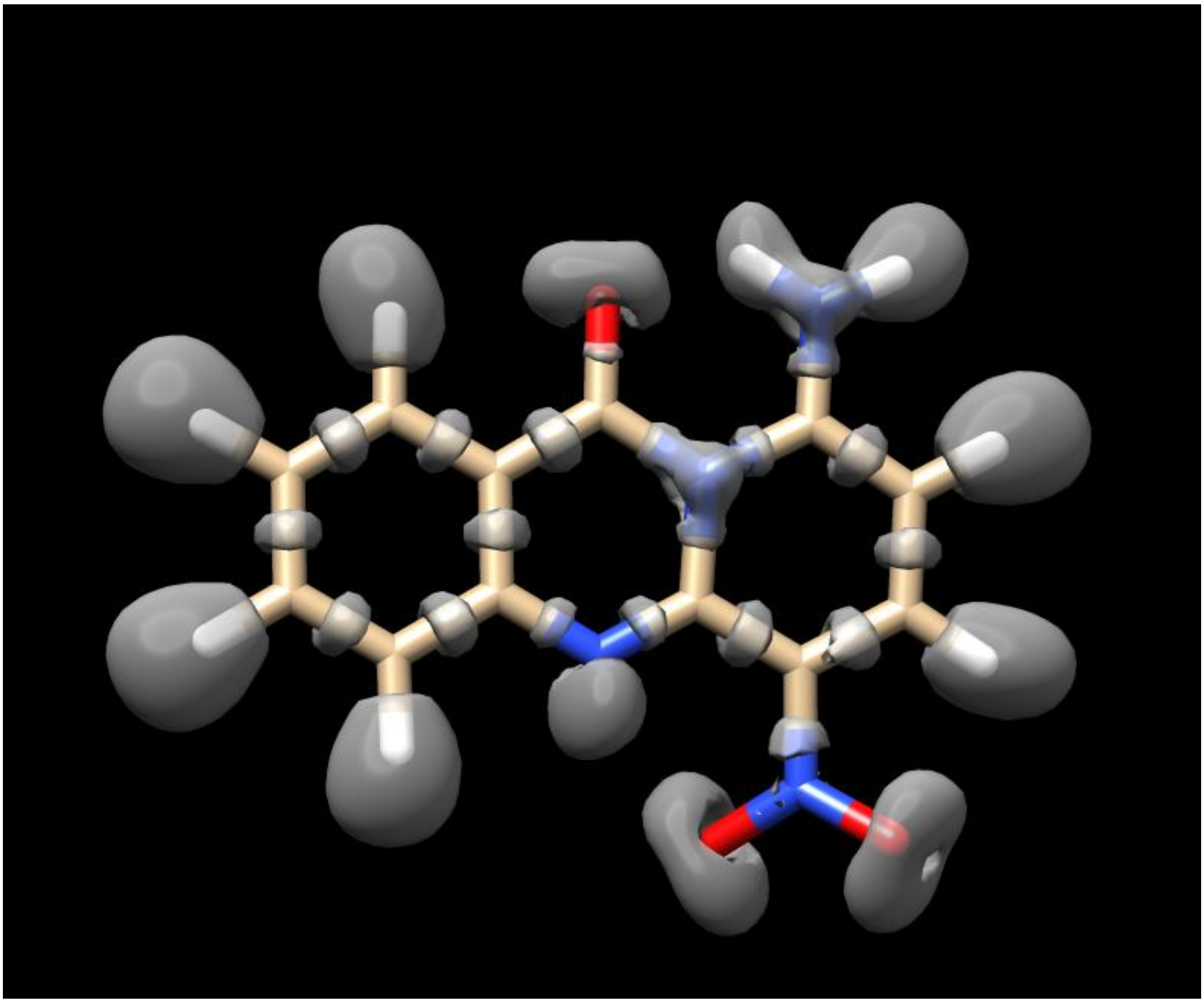

Figure S2a Electron localization function ELF isosurfaces of the compound **25**.

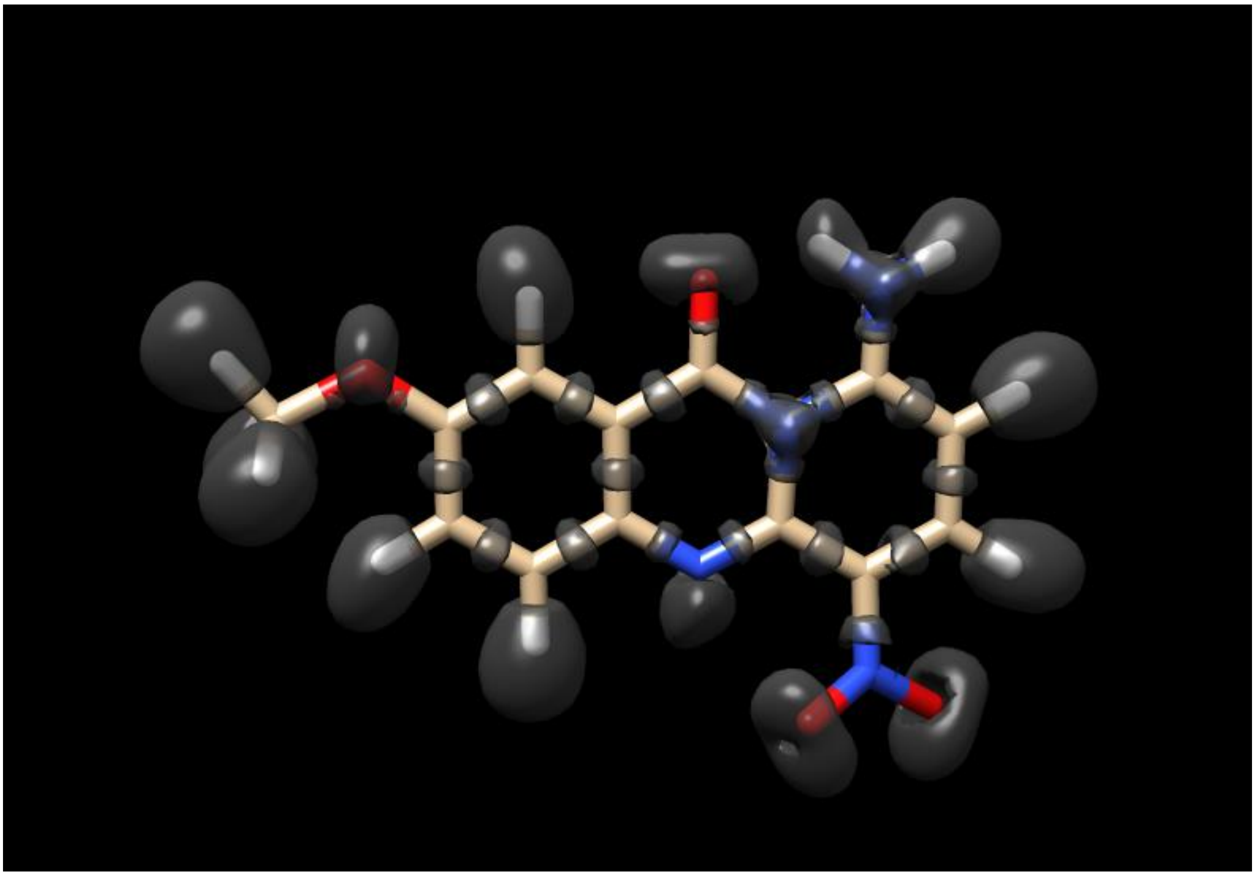

Figure S2b Electron localization function isosurfaces of the compound **29**.

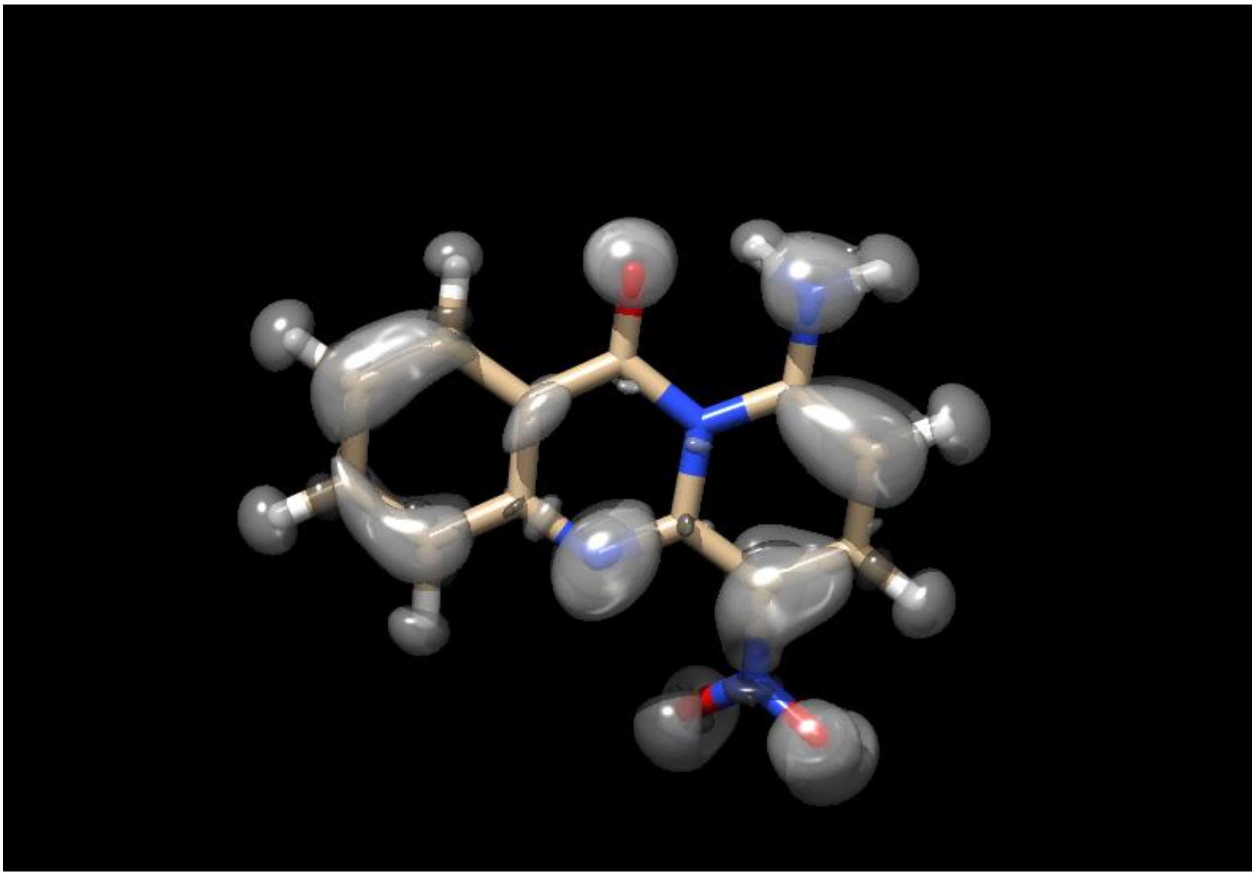

Figure S3a Fukui electrophilicity function f- function isosurface for compound **25**.

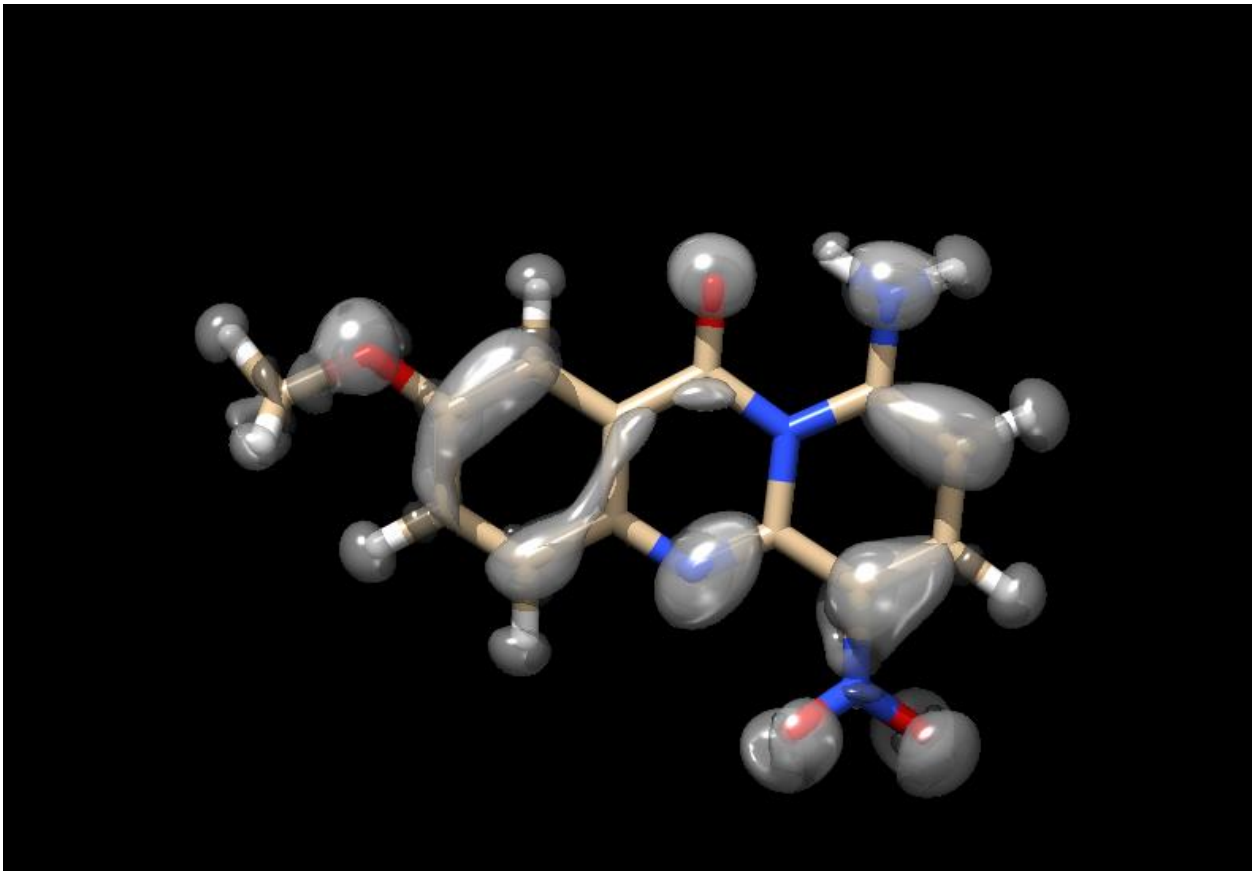

Figure S3b Fukui electrophilicity function f- function isosurface for compound **29**.

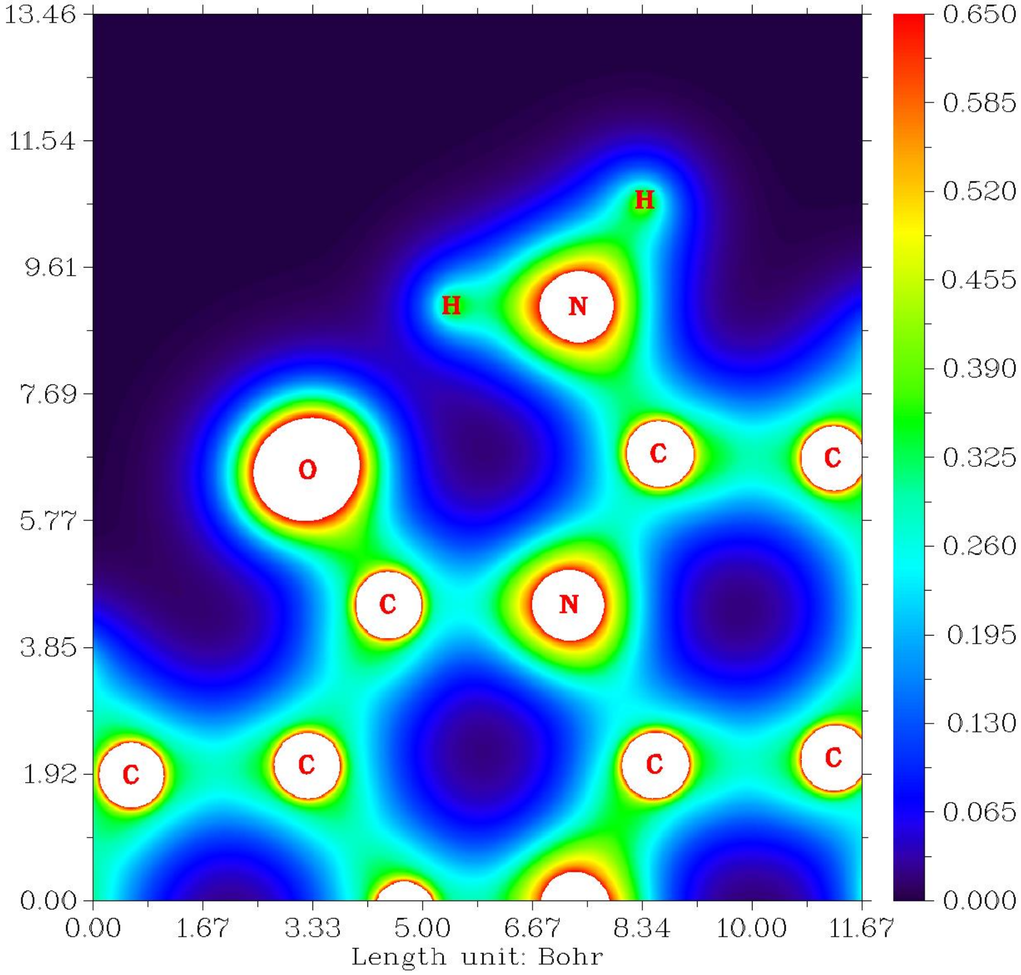

Figure S4a QTAIM of the Rho in the amide plane of compound **25**

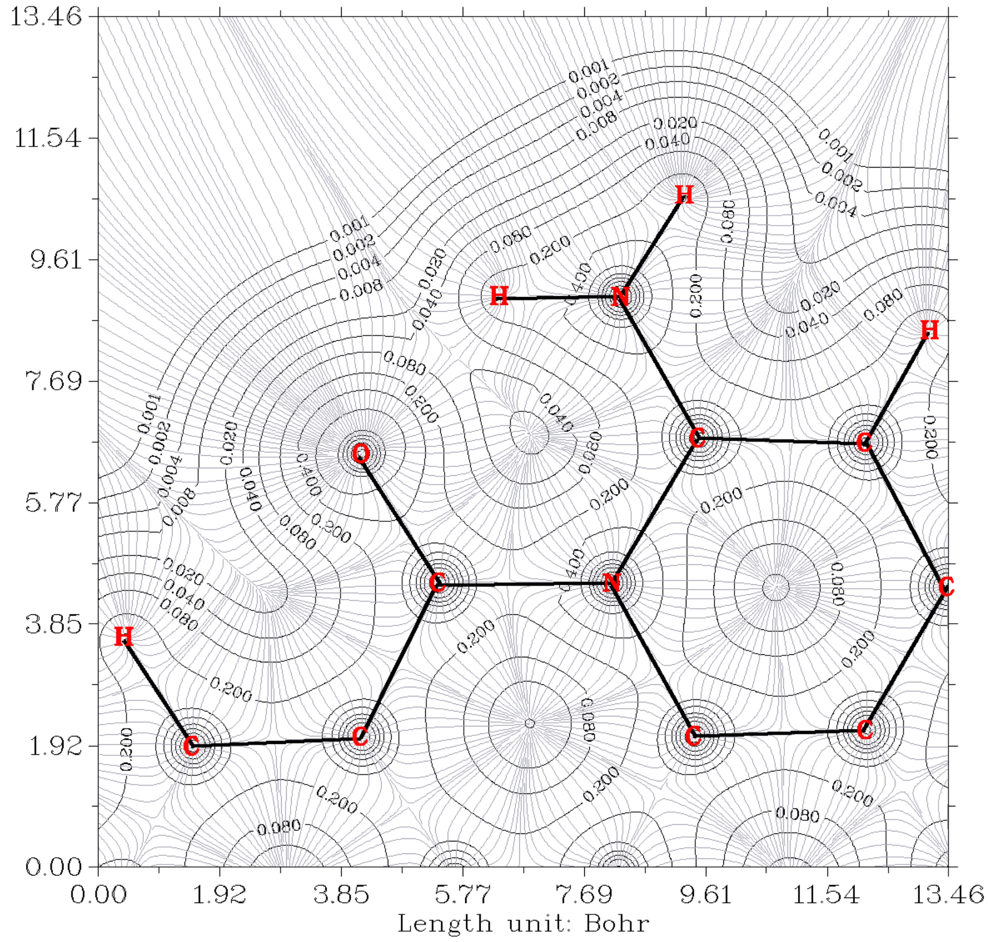

Figure S4b QTAIM of the Rho in the amide plane of compound **25** with the isovalues made visible

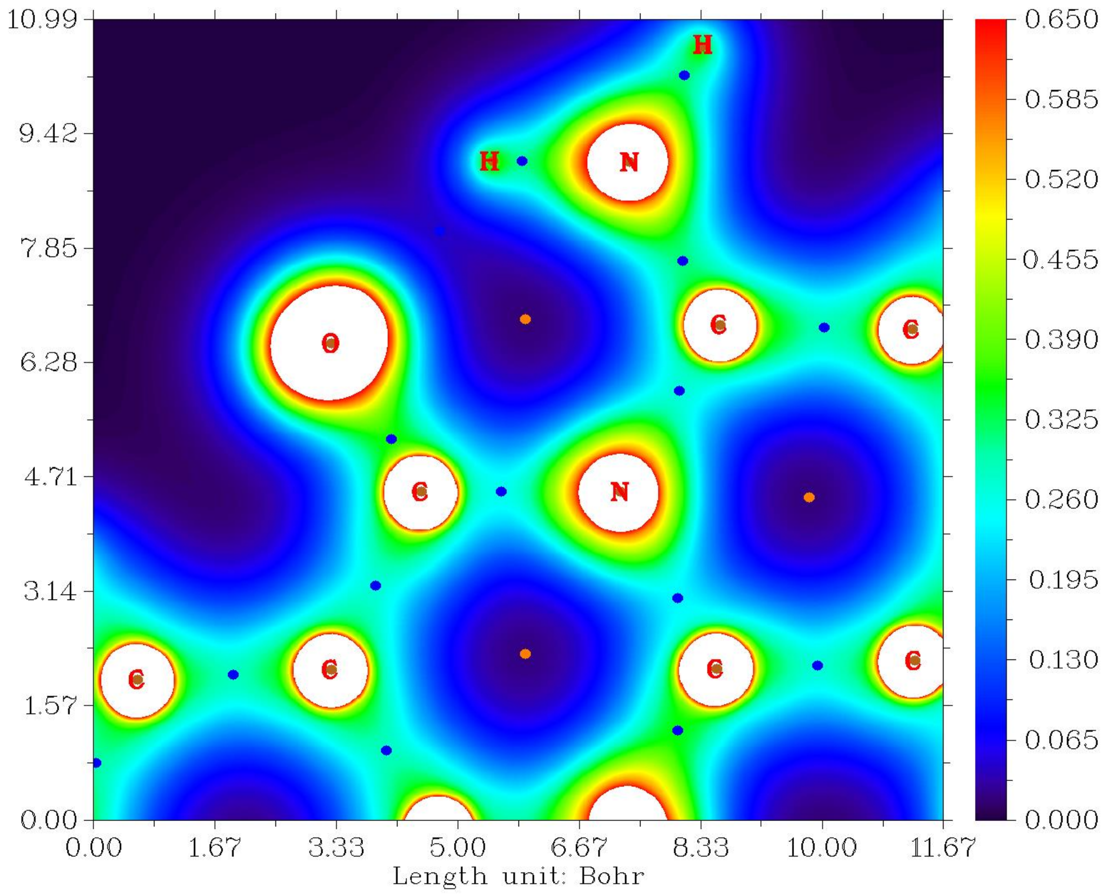

Figure S4c QTAIM of the Rho in the amide plane of compound **29**

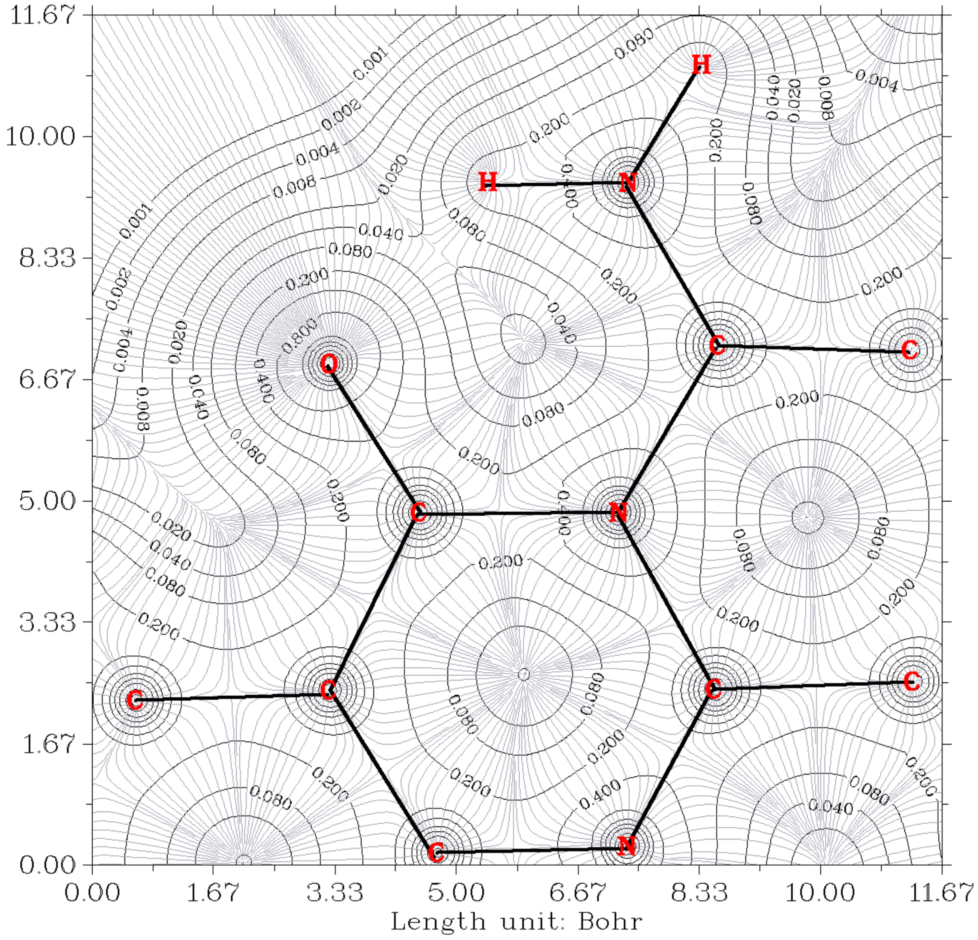

Figure S4d QTAIM of the Rho in the amide plane of compound **29** with the isovalues made visible

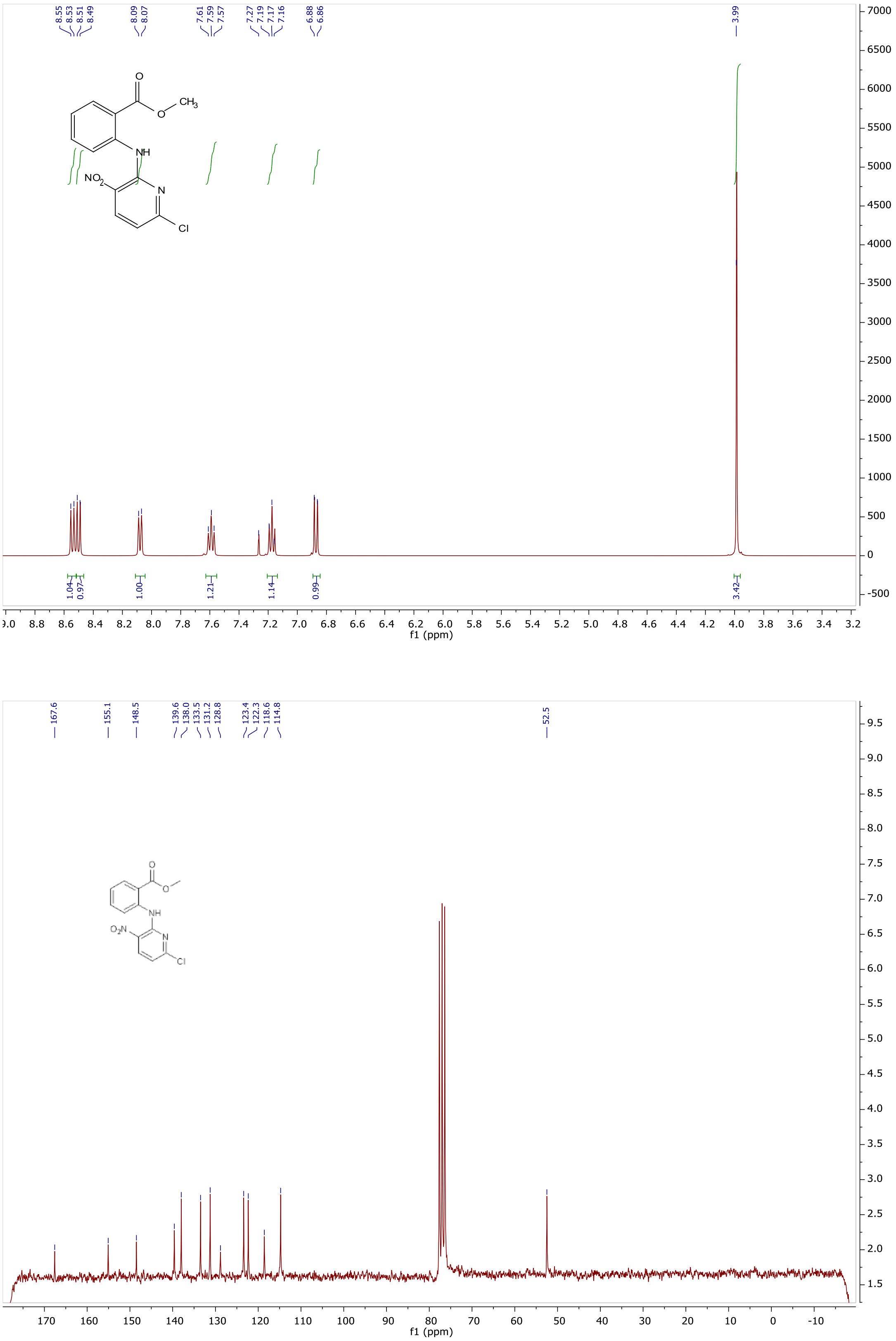

Figure S5: ^{1}H NMR and ^{13}C NMR spectrum of compound **4**.

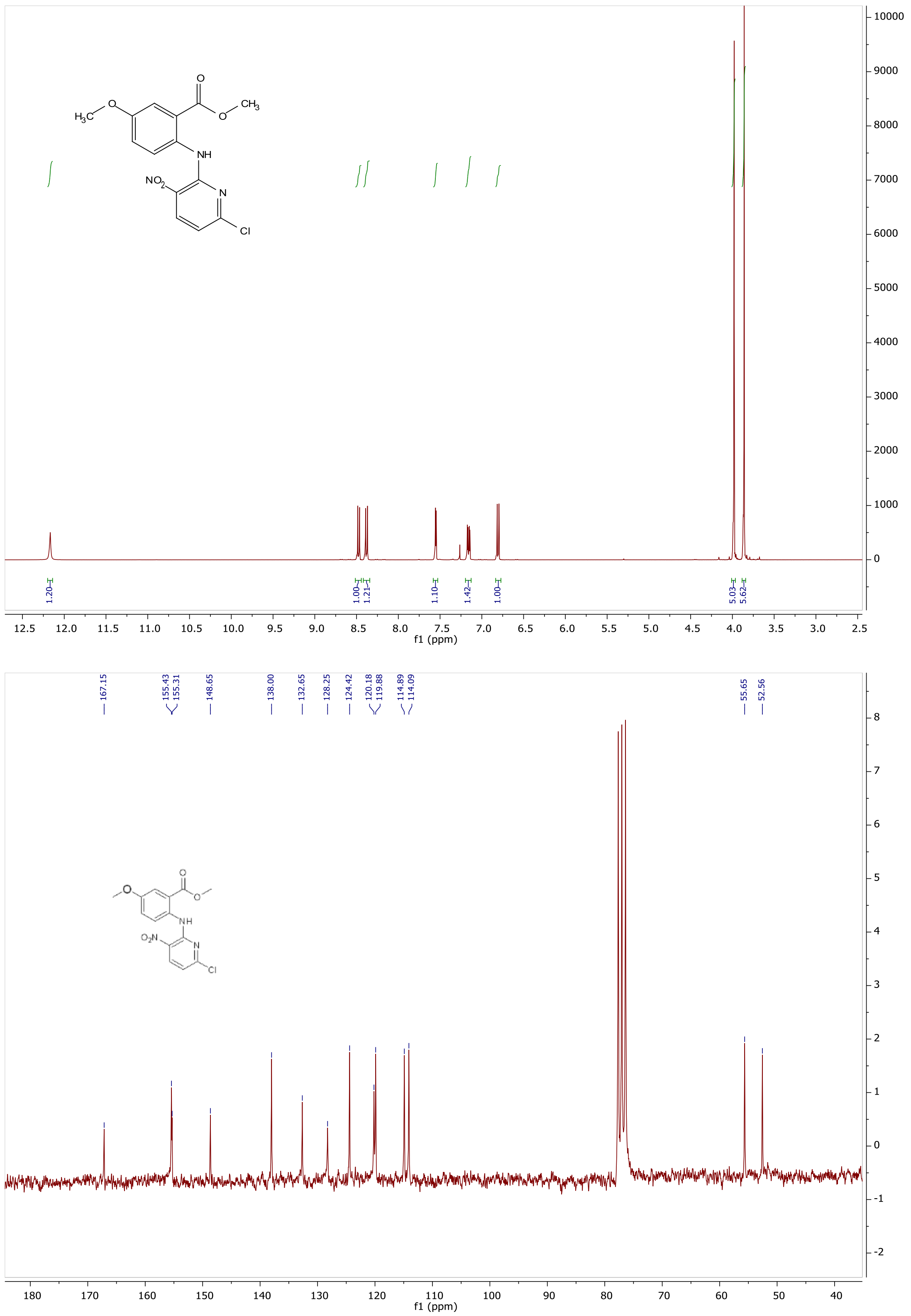

Figure S6: ^{1}H NMR and ^{13}C NMR spectrum of compound **5**.

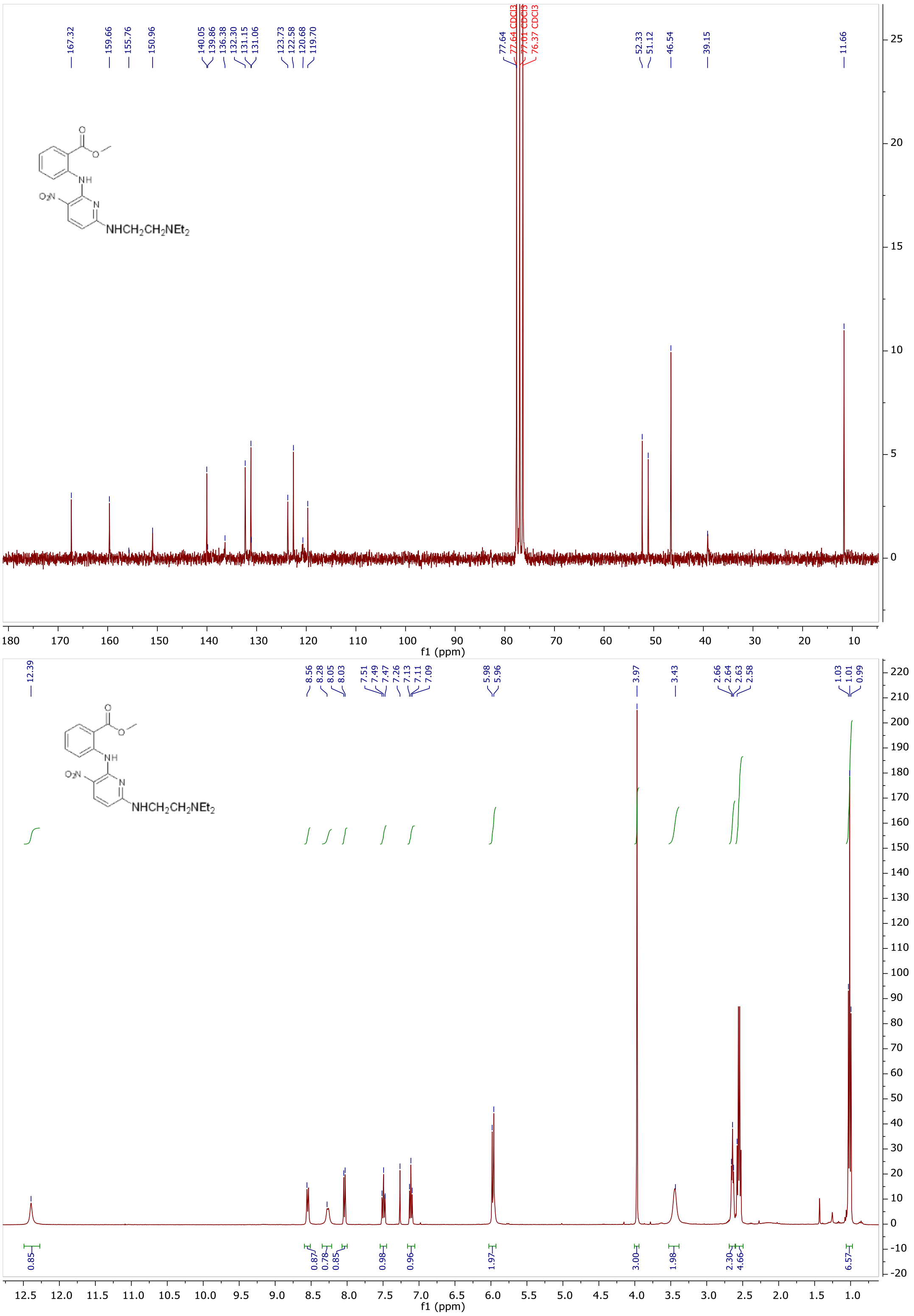

Figure S7: ^1H NMR and ^{13}C NMR spectrum of compound **6**.

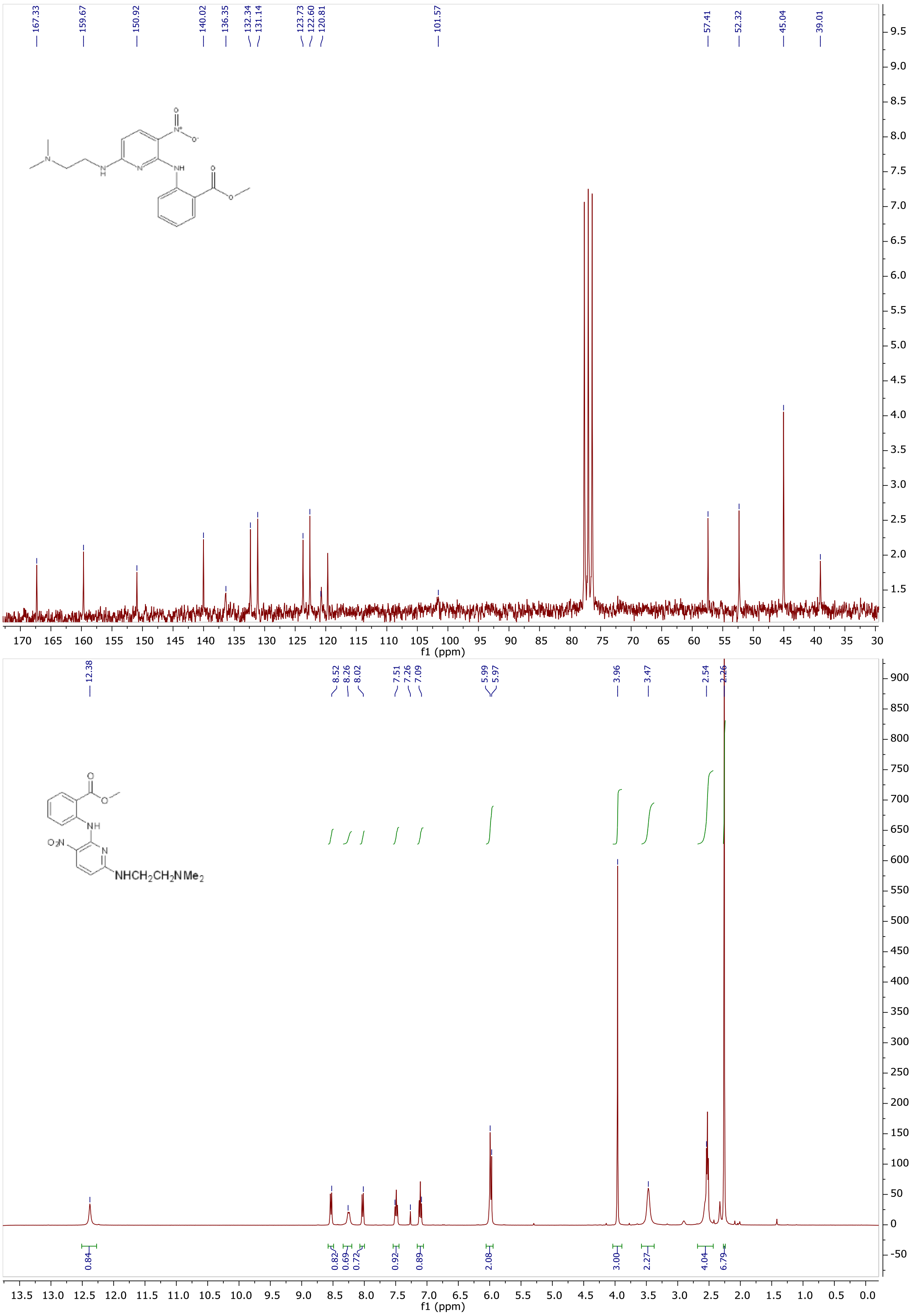

Figure S8: ^{1}H NMR and ^{13}C NMR spectrum of compound **7**.

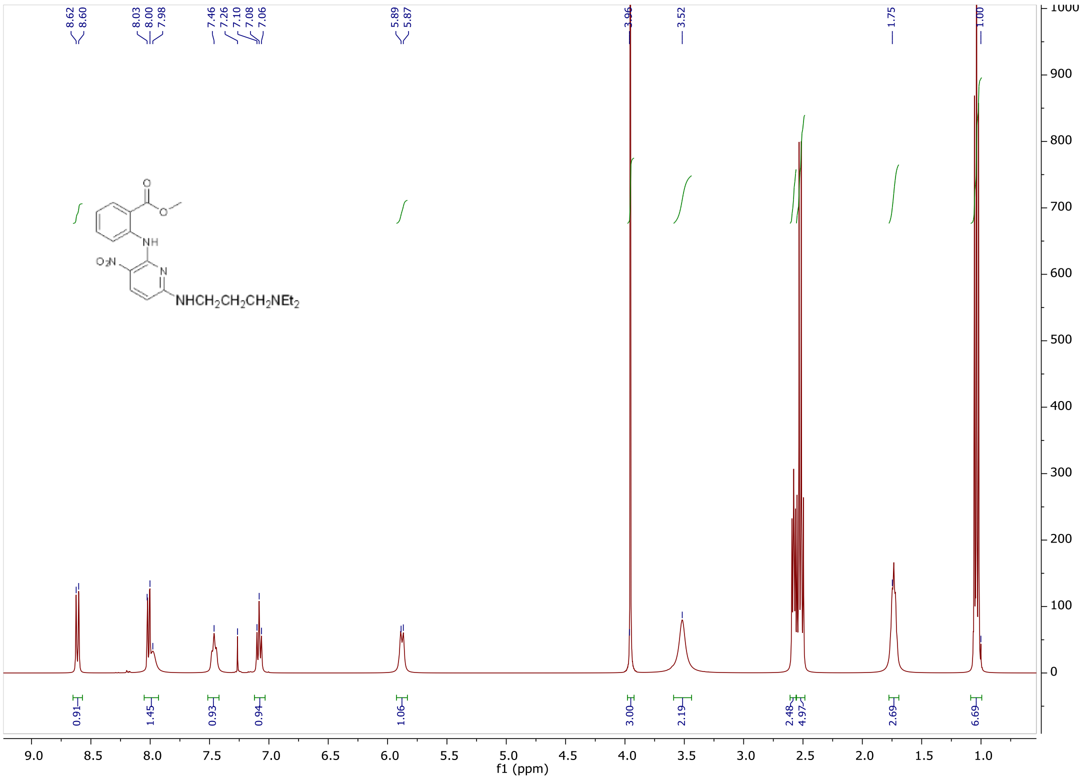

Figure S9: ^{1}H NMR and ^{13}C NMR spectrum of compound **8**.

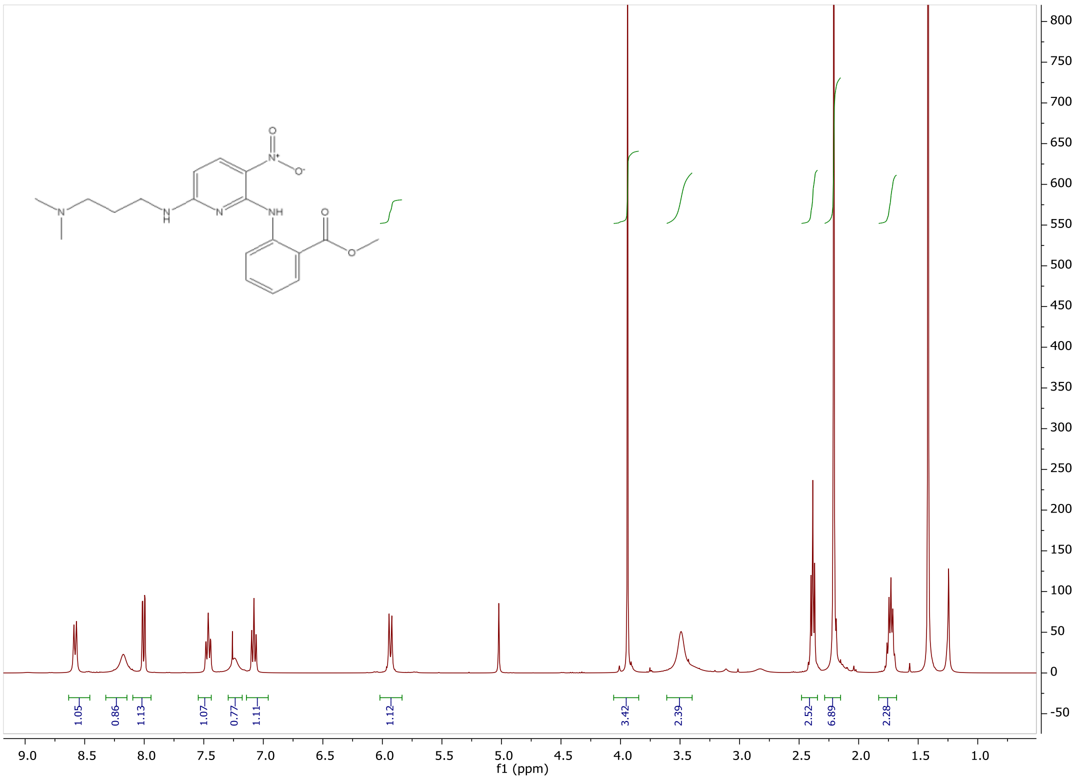

Figure S10: ^{1}H NMR and ^{13}C NMR spectrum of compound **9**.

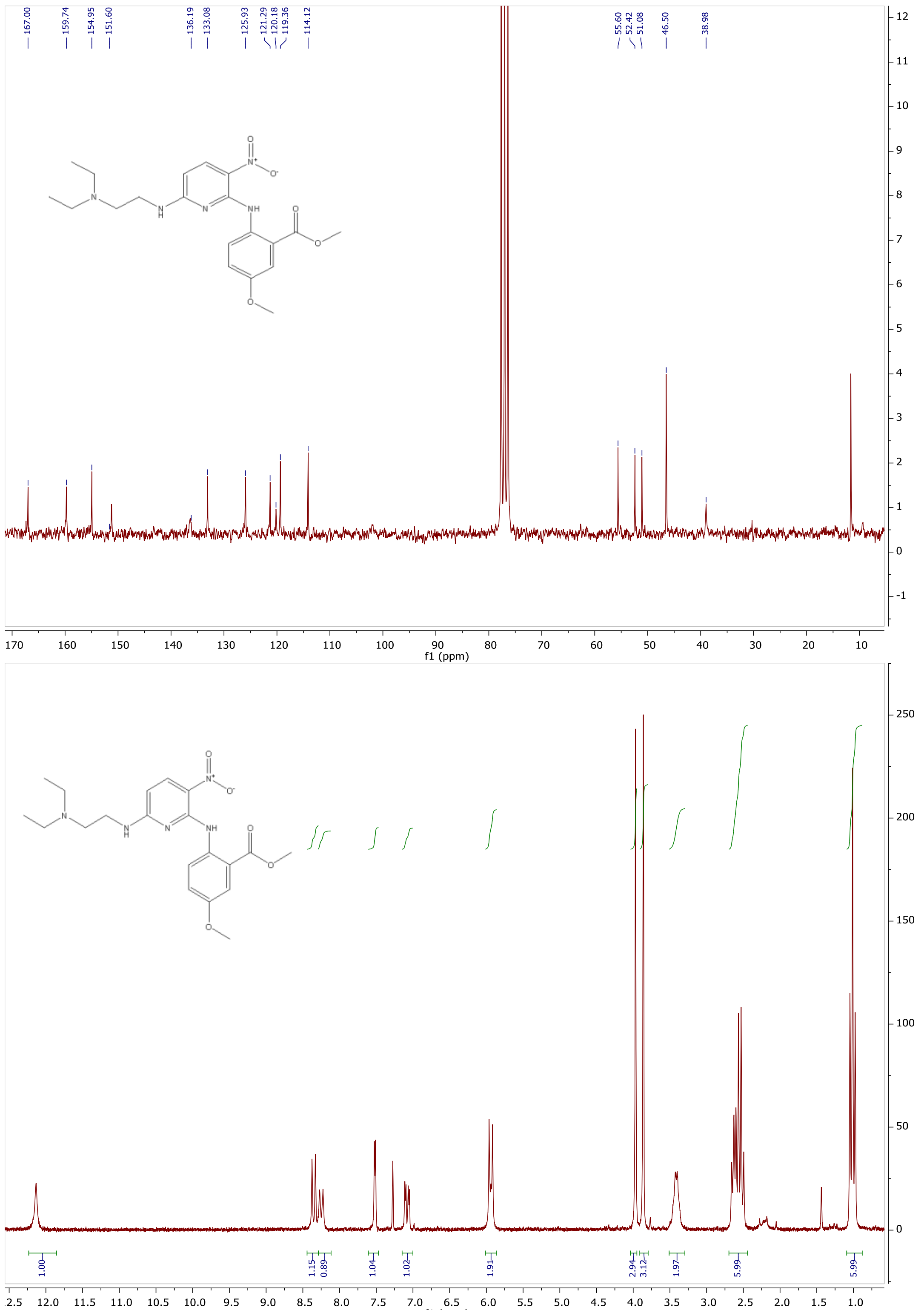

Figure S11: ^{1}H NMR and ^{13}C NMR spectrum of compound **10**.

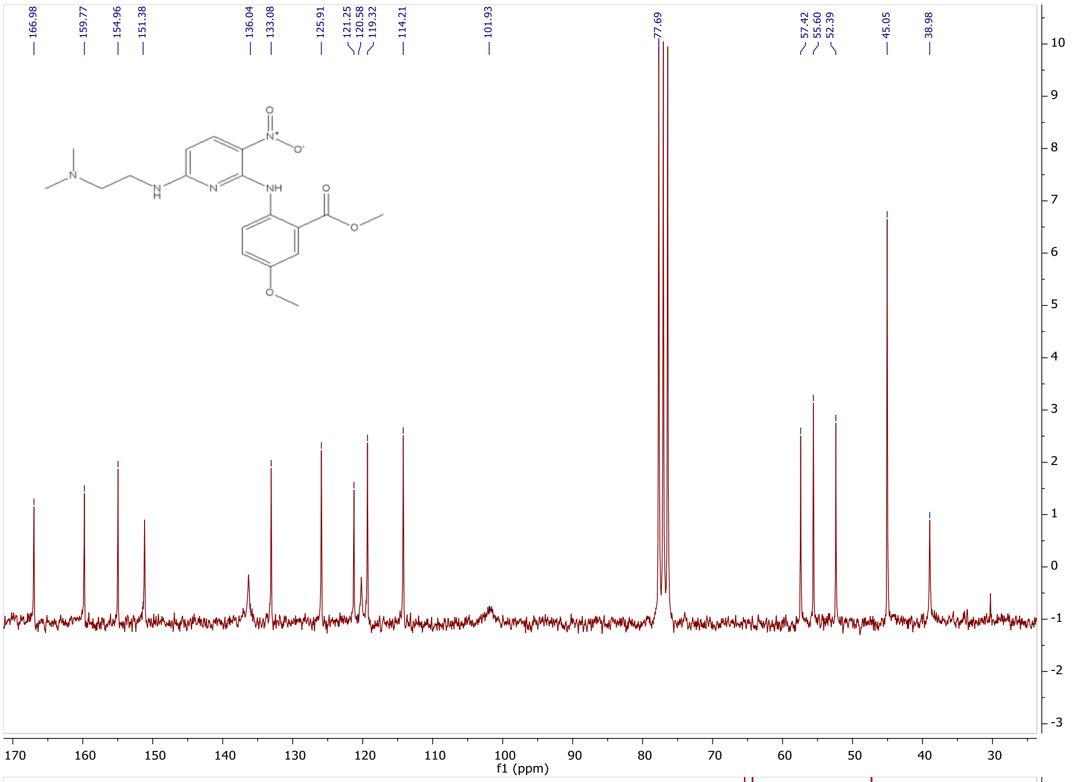

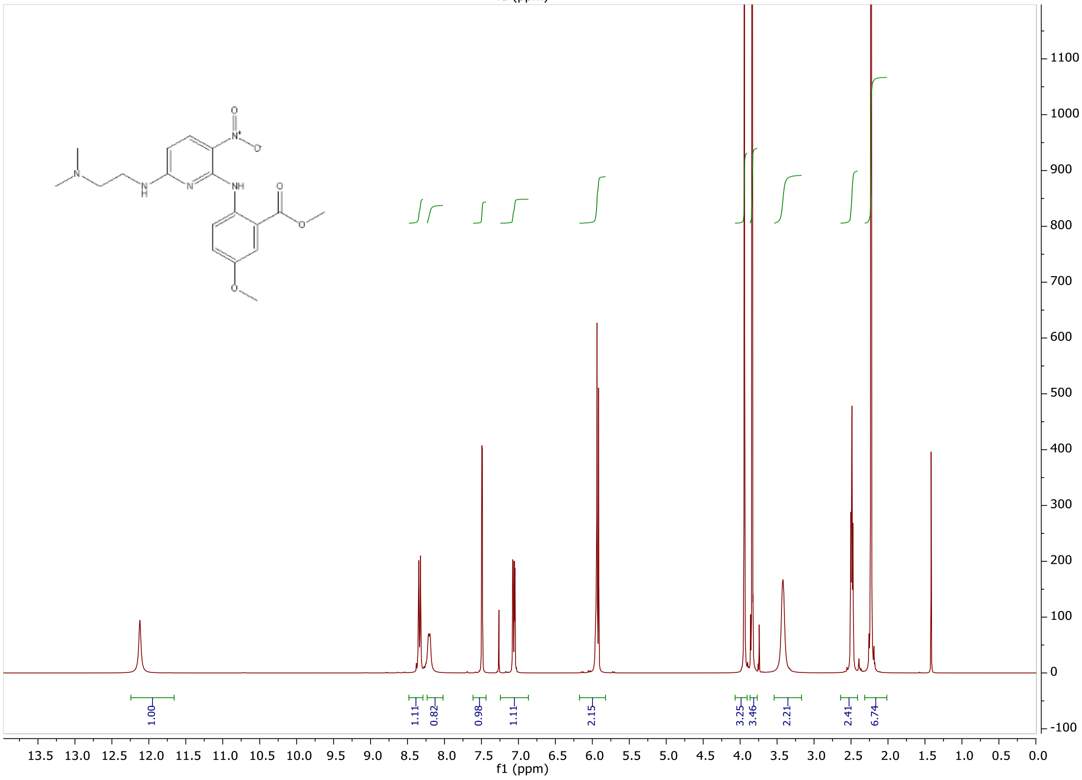

Figure S12: ^{1}H NMR and ^{13}C NMR spectrum of compound **11**.

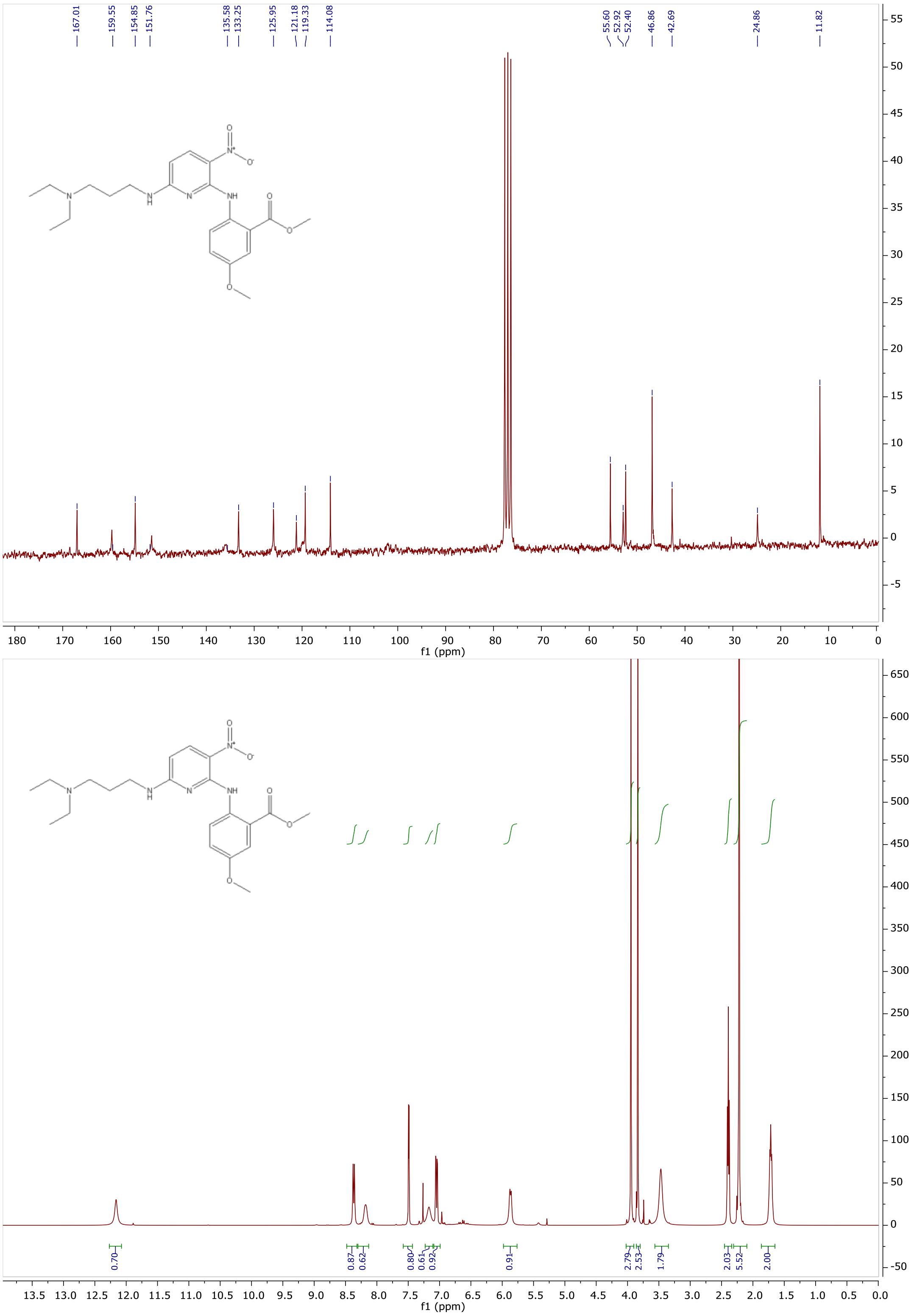

Figure S13: ^{1}H NMR and ^{13}C NMR spectrum of compound **12**.

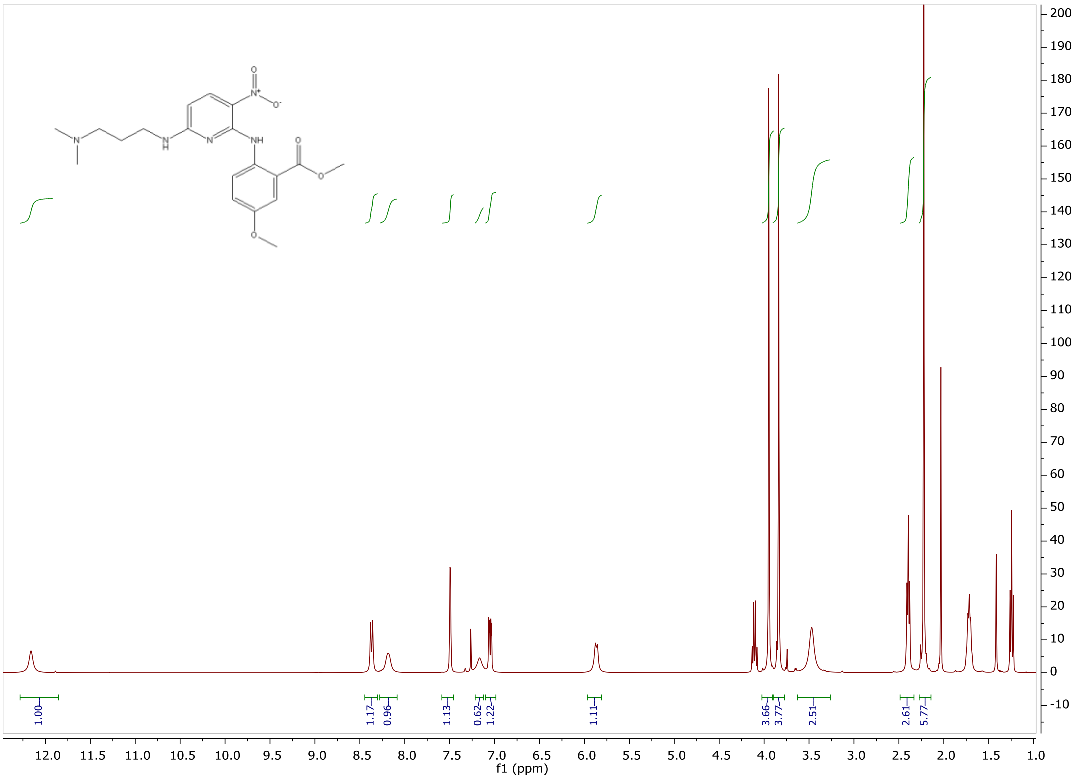

Figure S14: ^{1}H NMR and ^{13}C NMR spectrum of compound **13**.

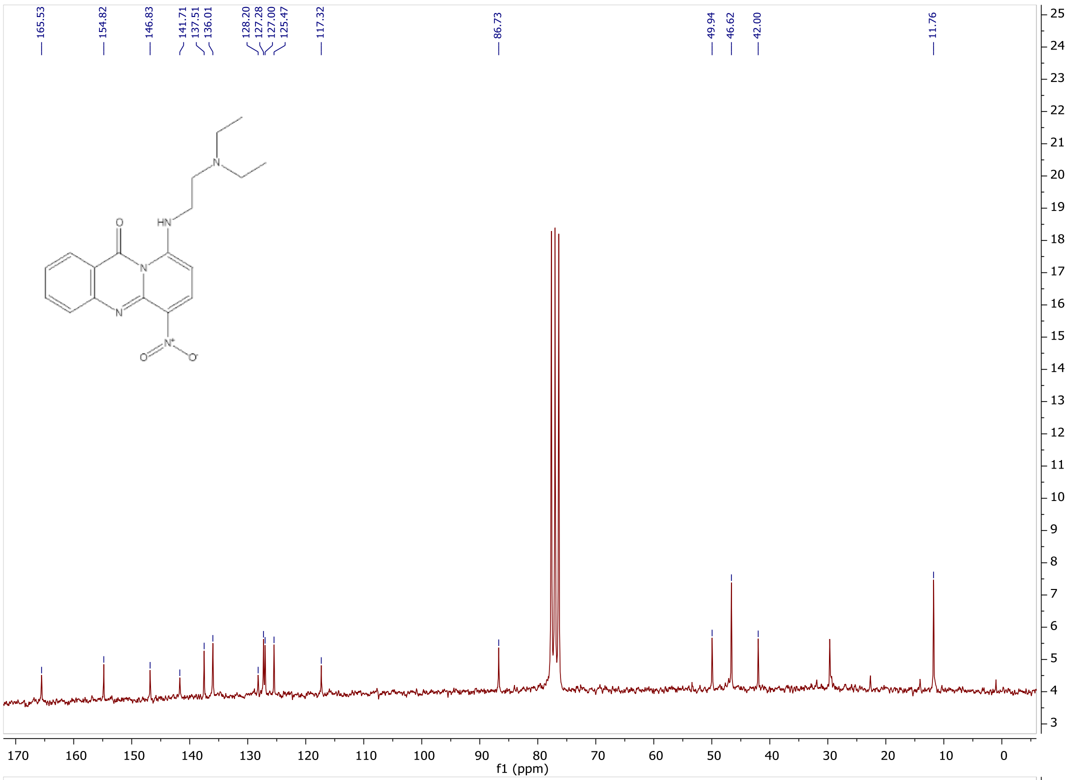

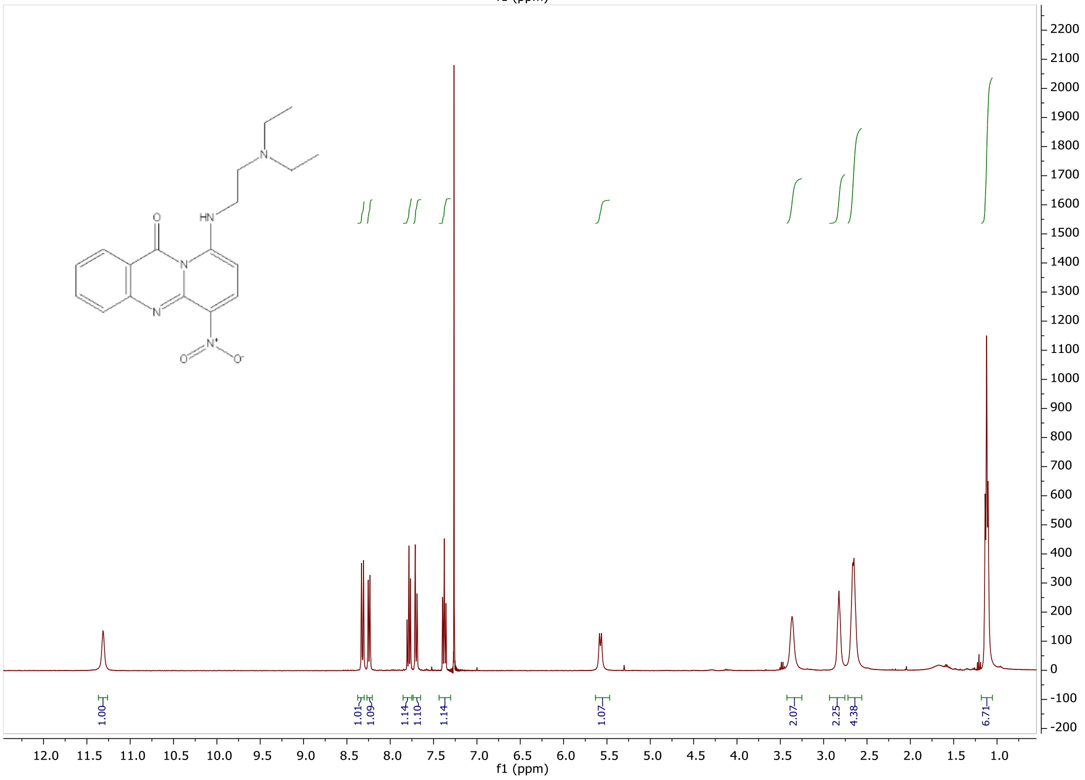

Figure S15: ^{1}H-NMR and ^{13}C NMR spectrum of compound **22**.

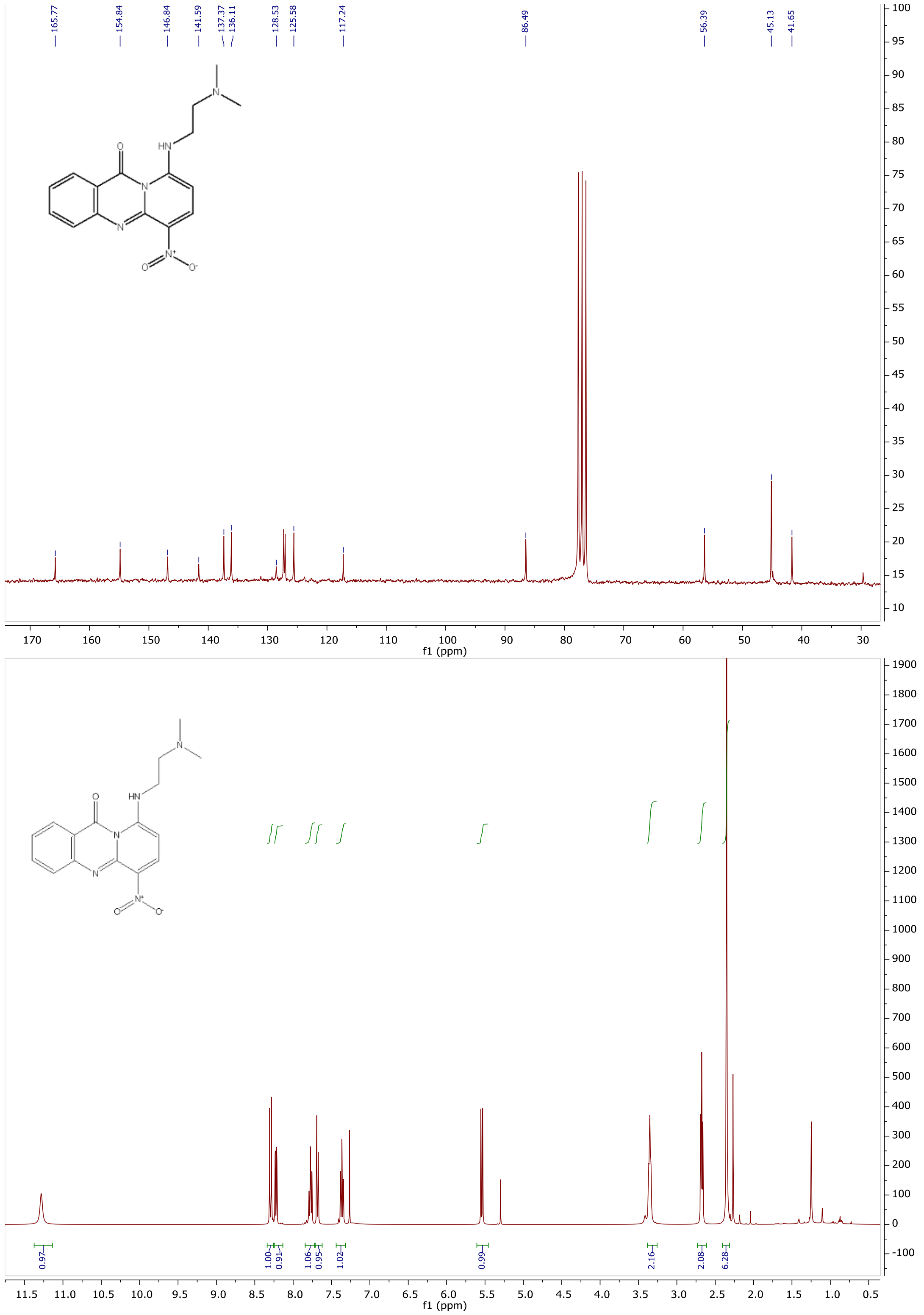

Figure S16: ^{1}H NMR and ^{13}C NMR spectrum of compound **23**.

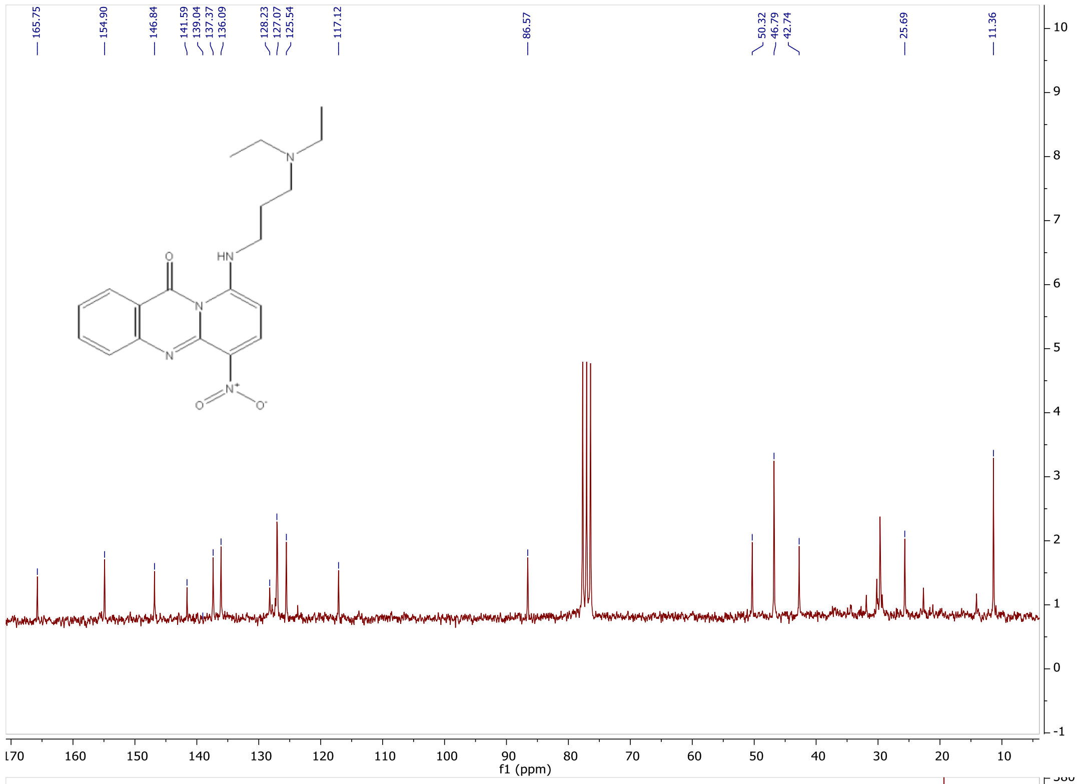

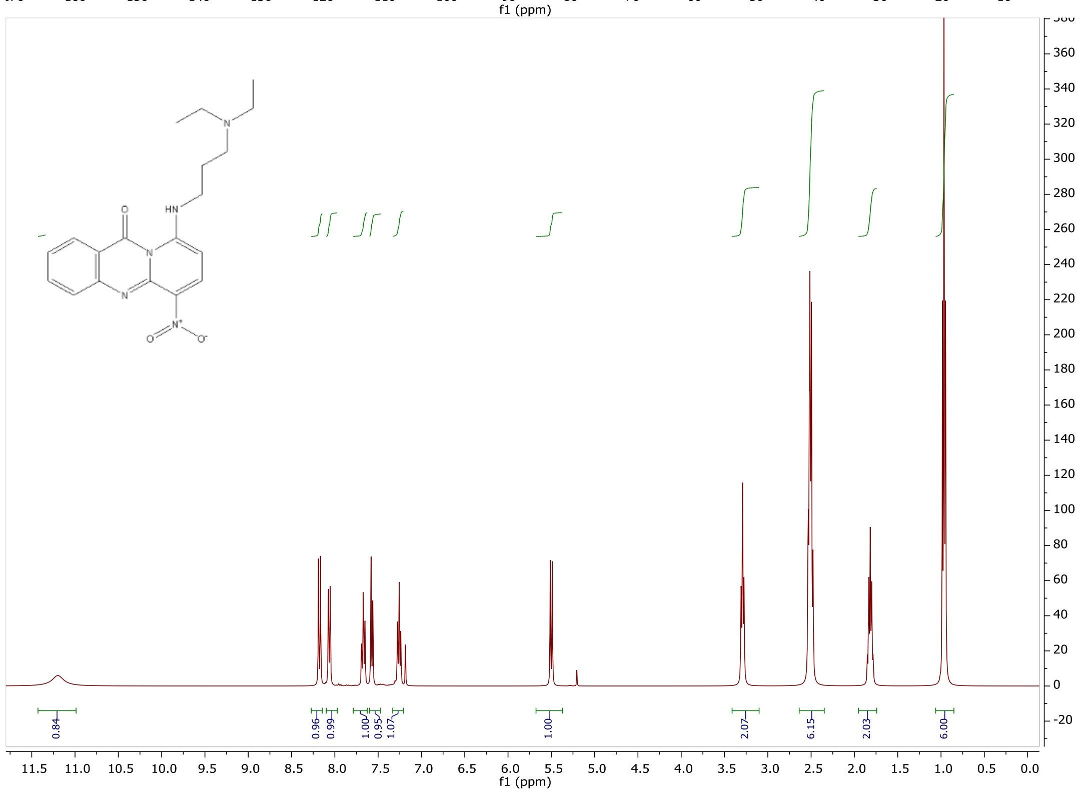

Figure S17: ^{1}H NMR and ^{13}C NMR spectrum of compound **24**.

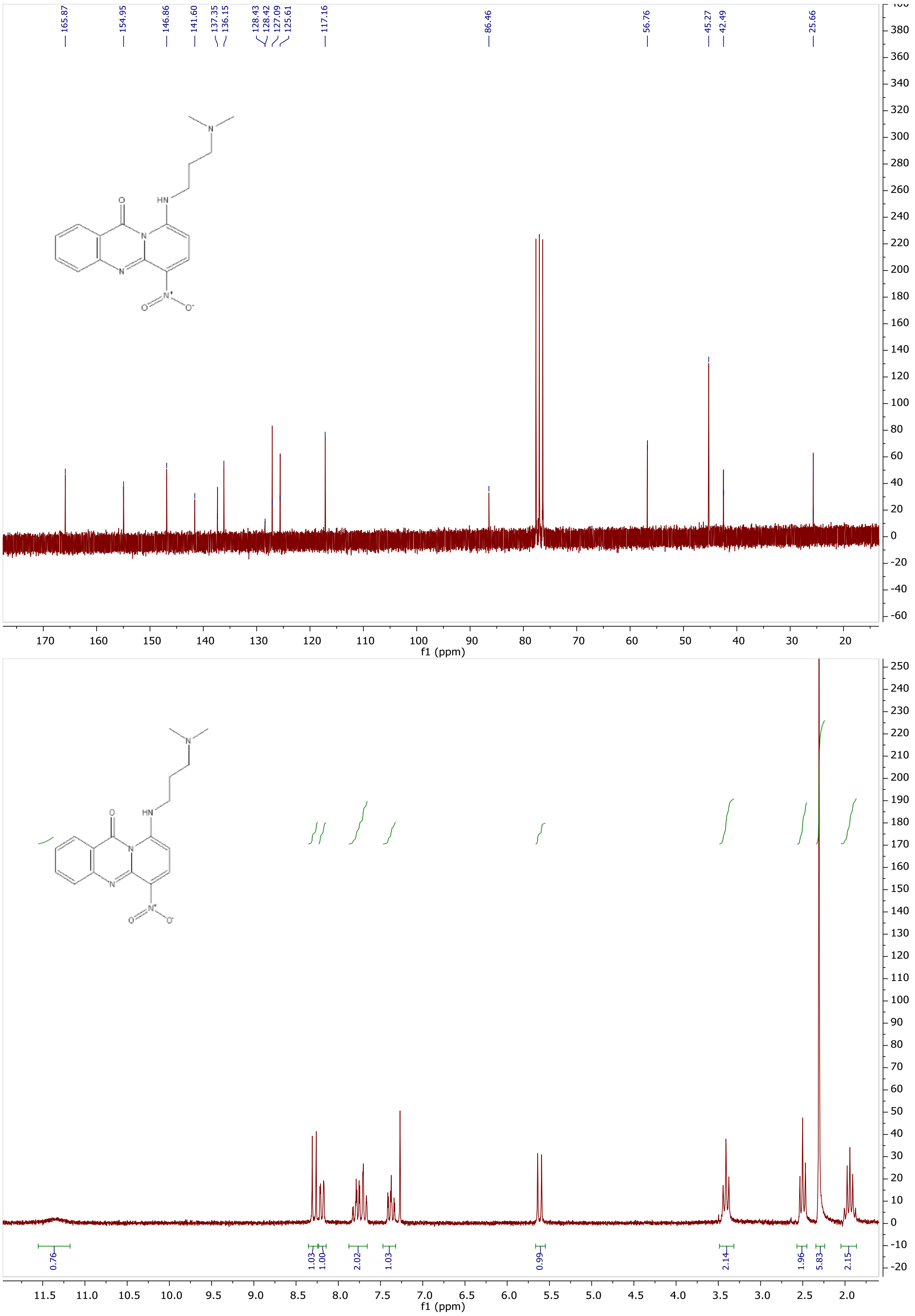

Figure S18: ^{1}H NMR and ^{13}C NMR spectrum of compound **25**.

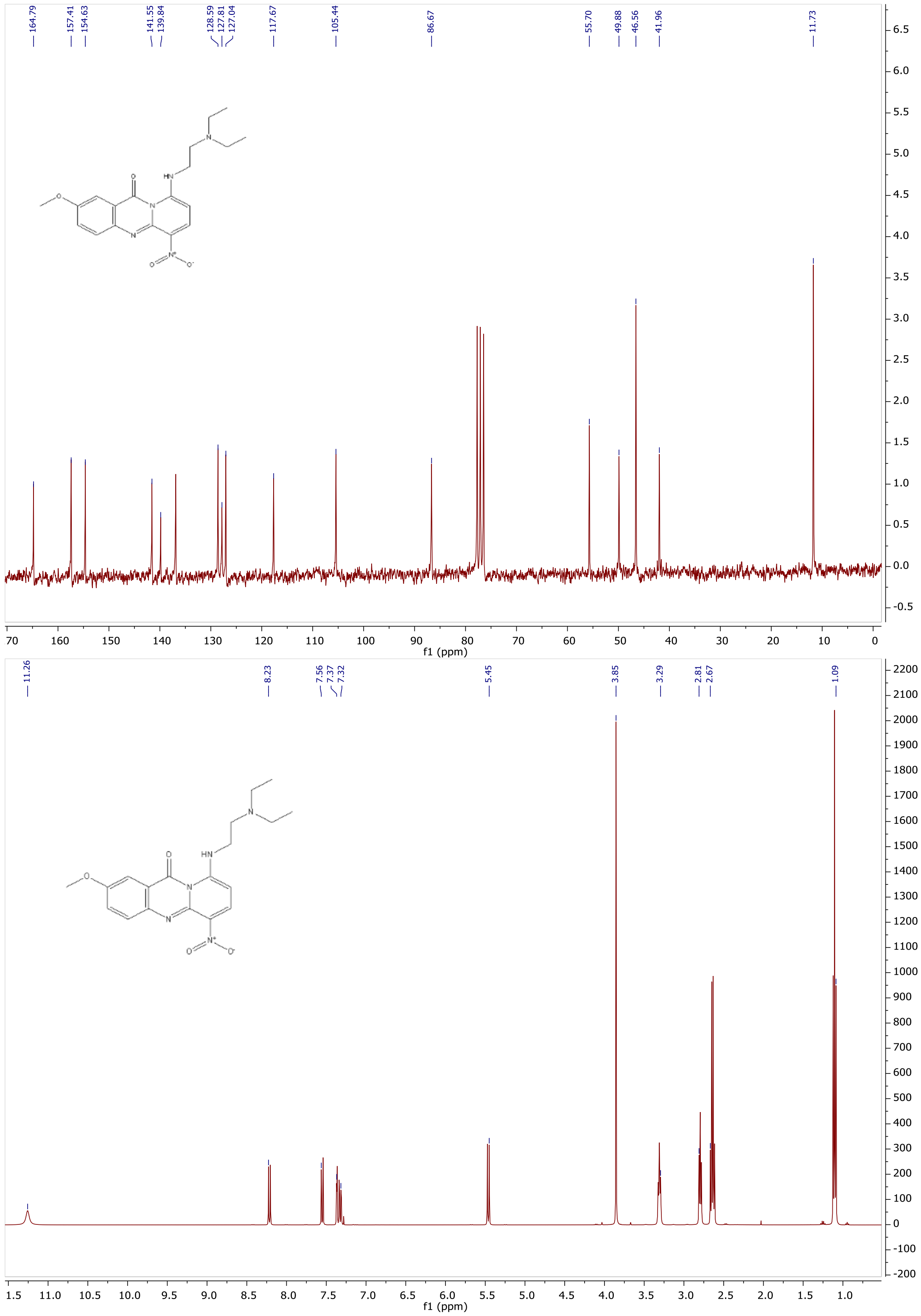

Figure S19: ^{1}H NMR and ^{13}C NMR spectrum of compound **26**.

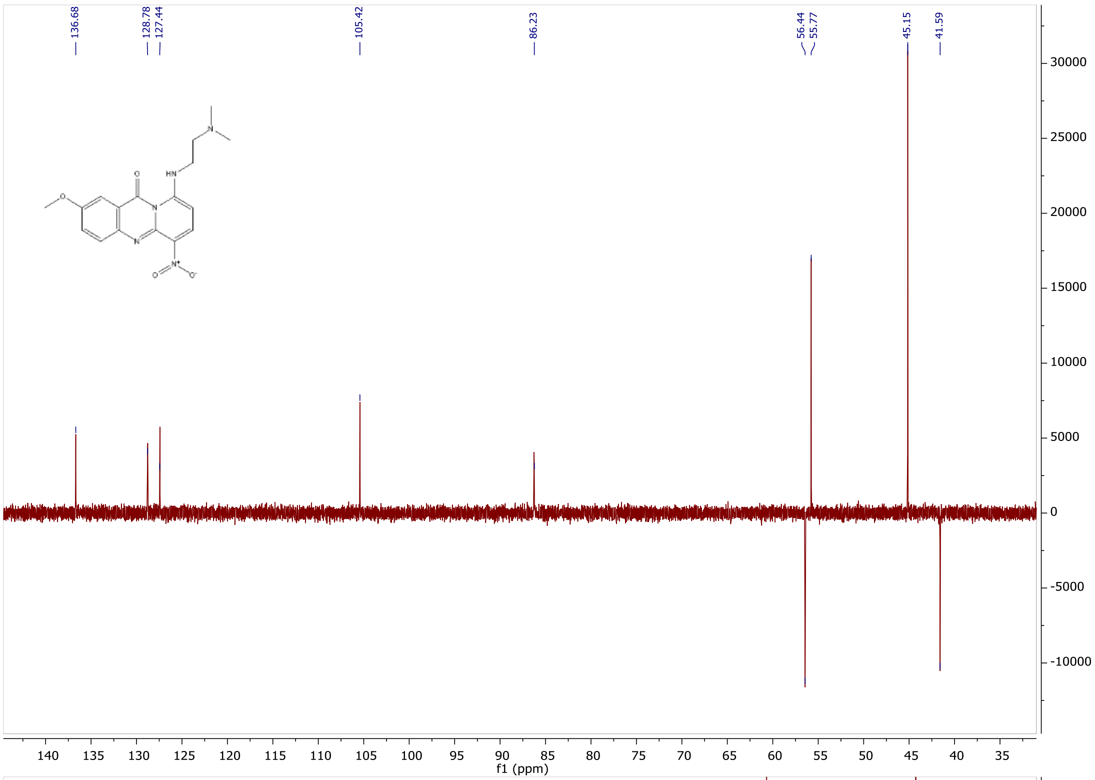

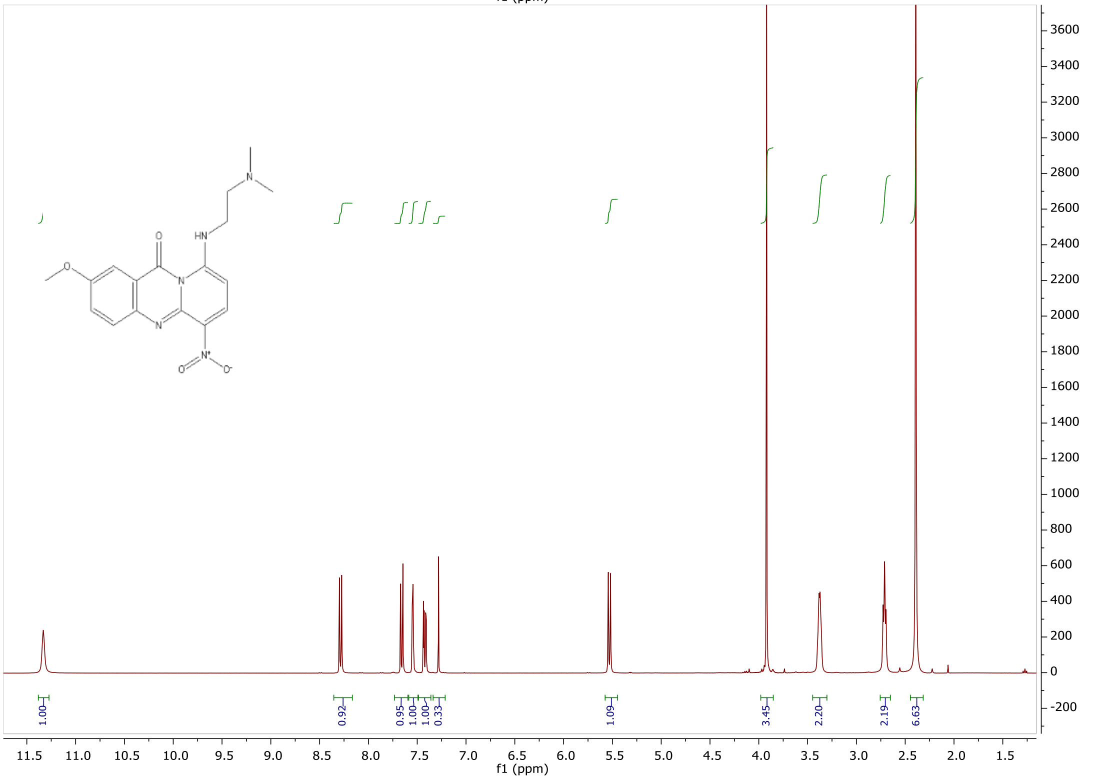

Figure S20: ^{1}H NMR and ^{13}C NMR spectrum of compound **27**.

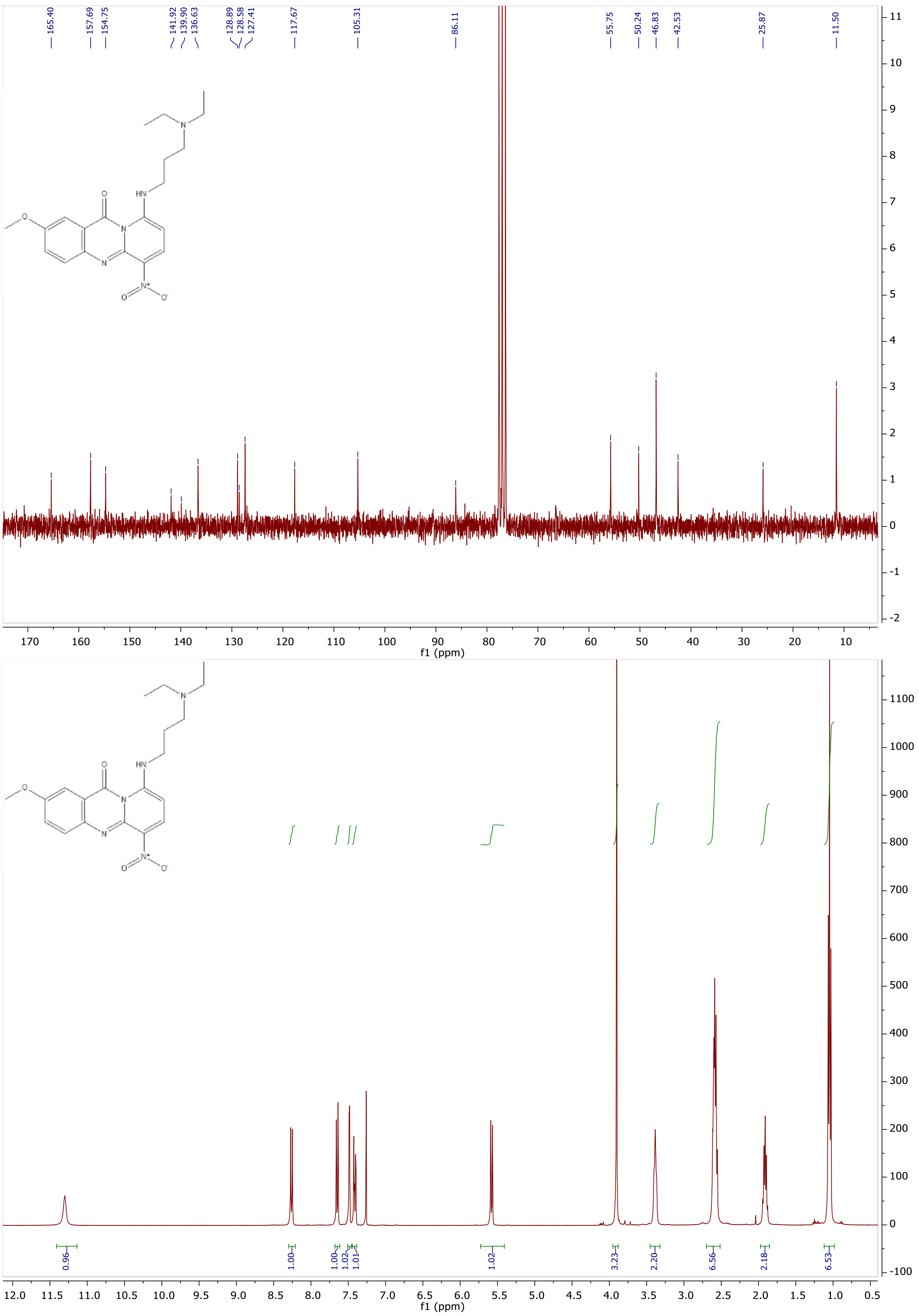

Figure S21: ^{1}H NMR and ^{13}C NMR spectrum of compound **28**.

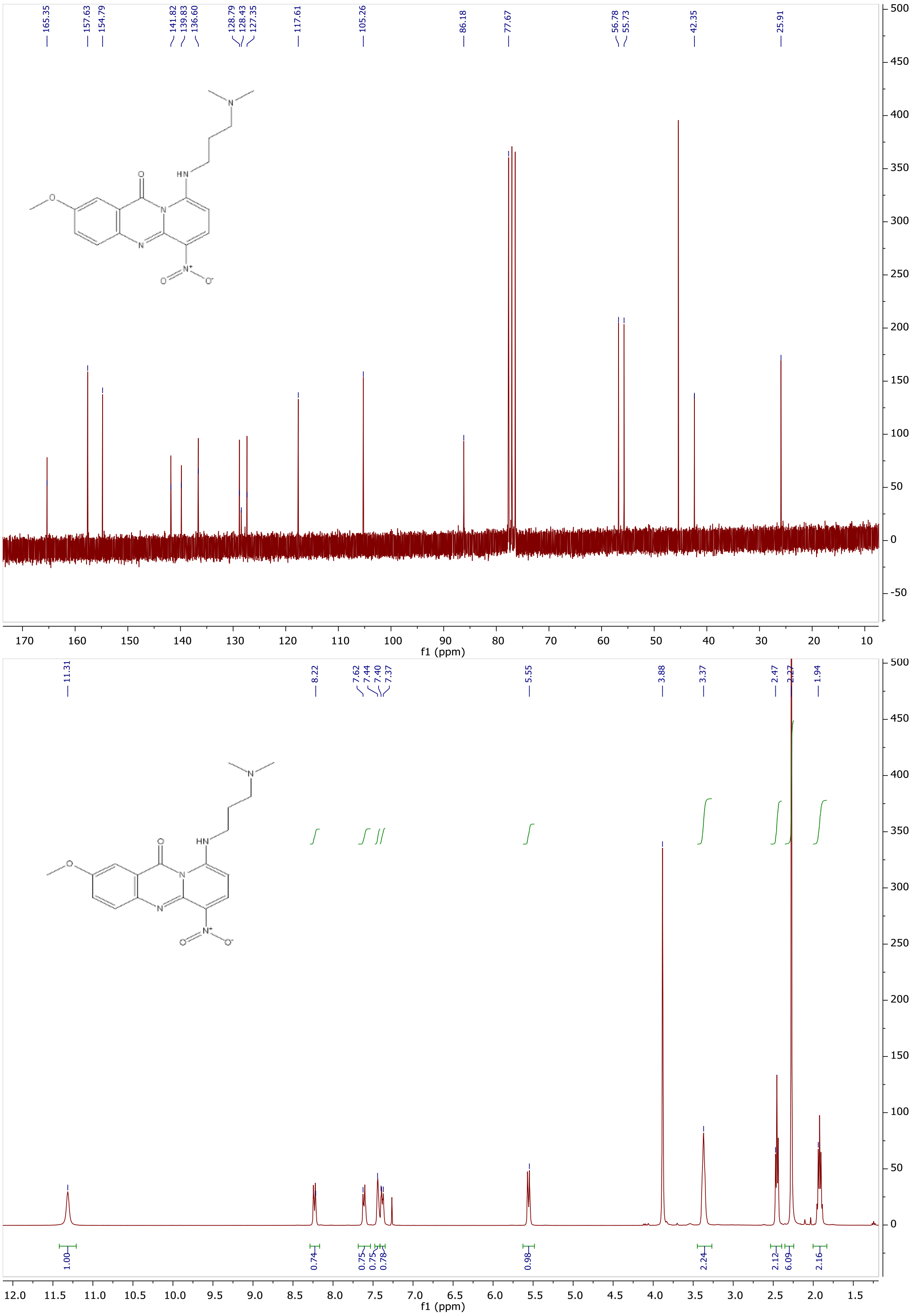

Figure S22: ^{1}H NMR and ^{13}C NMR spectrum of compound **29**.

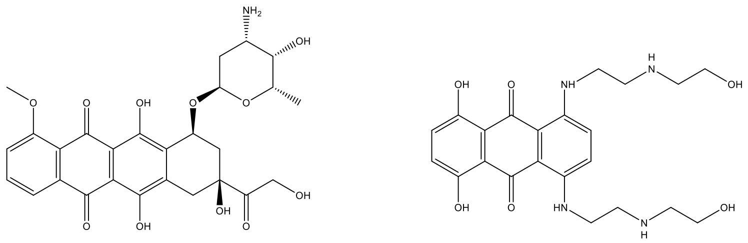

Doxorubicin Mitoxantrone

Figure S23: Structures of positive controls
